# Regulation of Superoxide by BAP31 through Its Effect on p22^phox^ and Keap1/Nrf2/HO-1 Signaling Pathway in Microglia

**DOI:** 10.1155/2021/1457089

**Published:** 2021-03-09

**Authors:** Xia Liu, Qing Yuan, Guo-xun Li, Cong-cong Jia, Jing-yu Liu, Yan-qiu Yang, Xiao-yu Wang, Yue Hou, Bing Wang

**Affiliations:** College of Life and Health Science, Northeastern University, 195 Chuangxin Road, Hunnan District, Shenyang, Liaoning Province 110819, China

## Abstract

Reactive oxygen species (ROS) production by activation of microglia is considered to be a major cause of neuronal dysfunction, which can lead to damage and death through direct oxidative damage to neuronal macromolecules or derangement of neuronal redox signaling circuits. BAP31, an integral ER membrane protein, has been defined as a regulatory molecule in the CNS. Our latest studies have found that BAP31 deficiency leads to activation of microglia. In this study, we discovered that BAP31 deficiency upregulated LPS-induced superoxide anion production in BV2 cells and mice by upregulating the expression level of p22^phox^ and by inhibiting the activation of Nrf2-HO-1 signaling. Knockdown of p22^phox^/keap1 or use of an NADPH oxidase inhibitor (apocynin) reversed the production of superoxide anion and inflammatory cytokines, which then reduced neuronal damage and death *in vitro* and *in vivo*. These results suggest that BAP31 deficiency contributes to microglia-related superoxide anion production and neuroinflammation through p22^phox^ and keap1. Furthermore, the excess superoxide anion cooperated with inflammatory cytokines to induce the damage and death of neurons. Thus, we determined that BAP31 is an important regulator in superoxide anion production and neuroinflammation, and the downstream regulators or agonists of BAP31 could therefore be considered as potential therapeutic targets in microglial-related superoxide anion production and neuroinflammation.

## 1. Introduction

Neurodegenerative diseases including Alzheimer's disease (AD), Parkinson's disease (PD), and multiple sclerosis (MS) are characterized by oxidative damage, chronic neuroinflammation, neuronal degeneration, and death in specific regions of the central nervous system (CNS) [1–3]. Reactive oxygen species (ROS), including the superoxide anion (O_2_^•-^), hydrogen peroxide (H_2_O_2_), and hydroxyl radical (OH^−^), are the natural by-products of aerobic metabolism. O_2_^•-^, which is the precursor for most other ROS, can be catalyzed into H_2_O_2_ by superoxide dismutases (SODs) and further reduced to the hydroxyl radical or water by peroxidases. ROS are well-known stress signaling molecules in cells, and can be increased dramatically by environmental stress and disease [4]. The formation of superoxide anion and hydrogen peroxide is one of several common mechanisms that are described as triggers of neurodegeneration, with persistently unquenched levels of superoxide anion and hydrogen peroxide ultimately leading to oxidative damage to the lipids, proteins, and DNA essential for biological homeostatic states. Since the effects of oxidative stress are widespread and the brain has low antioxidant capacities, neurons are particularly vulnerable to oxidative damage induced by excess superoxide anion. In particular, superoxide anion production by microglia is considered to be a major cause of neuronal dysfunction, damage, and death through direct oxidative damage to neuronal macromolecules or derangement of neuronal redox signaling circuits, and the ROS-amplified proinflammatory response in microglia also drives neuropathology [5, 6].

The generation of superoxide anion and hydrogen peroxide is regulated by specific enzymes, such as NADPH oxidase (NOX). NOX is a multisubunit enzyme complex; under resting conditions, the different subunits of this complex are localized in the cytosol (p40^phox^, p47^phox^, and p67^phox^) and cellular membrane (p22^phox^ and gp91^phox^), while upon stimulation, the catalytically active complex is assembled in the plasma membrane [7, 8], resulting in superoxide anion and hydrogen peroxide production.

Nuclear factor erythroid 2-related factor 2 (Nrf2), a key regulator of endogenous defensive systems against oxidative stress, is produced by active microglia induced by oxidative stress in the brain [9]. Kelch-like ECH-associated protein 1 (keap1) is responsible for the cytosolic sequestration of Nrf2 under physiological conditions. Nrf2 is constitutively expressed in the cytoplasm and translocated into the nucleus under oxidative injury condition [10]. Heme oxygenase-1 (HO-1), a 32 kDa cytoprotective enzyme with strong immunomodulatory and anti-inflammatory properties, is positively regulated by Nrf2 [11]. Recent studies indicate that activation of Nrf2 signaling could be a popular strategy to prevent superoxide anion and inflammation-mediated neuronal toxicity [12, 13].

Superoxide anion and hydrogen peroxide produced by either microglia or the surrounding environment are currently considered not only to impact neurons but also to modulate microglial activity. They act as both a signaling molecule and a mediator of inflammation. LPS induces microglia to produce superoxide anion and hydrogen peroxide and several proinflammatory molecules such as TNF*α*, IL-1*β*, inducible nitric-oxide synthase, prostaglandin E2, and monocyte chemotactic protein-1 (MCP-1) via the NOX pathway.

B cell receptor-associated protein 31 (BAP31) is an endoplasmic reticulum (ER) membrane protein. Recently, BAP31 was found to be a regulatory molecule for immunity in the central nervous system [14]. BAP31 deficiency accelerates the formation of amyloid-*β* plaques in APP/PS1 mice [15] and exacerbates the activation of microglia and the death of neurons induced by LPS [16].

In this study, we investigated whether BAP31 influenced the superoxide anion production. Our findings implied that BAP31 deficiency accentuated LPS-induced superoxide anion and hydrogen peroxide production by both increasing the superoxide anion production by upregulating p22^phox^ expression and inhibiting superoxide anion scavenging through inhibiting the keap1/Nrf2/HO-1 signaling pathways. Knockdown of p22^phox^/keap1 or use of an inhibitor of NOX reversed these functions and protected neurons, indicating that BAP31 may be a key regulatory molecule in the nervous system by alleviating superoxide anion production in microglial cells.

## 2. Materials and Methods

### 2.1. Antibodies and Chemical Reagents

Dulbecco's modified Eagle's medium (DMEM), fetal bovine serum (FBS), and 0.25% trypsin were purchased from Gibco BRL (Grand Island, NY, USA). Anti-BAP31, anti-*β*-actin, anti-keap1, anti-Nrf2, anti-histone, anti-HO-1, anti-CD206, anti-CD86, and anti-8-OHdG were from Cell Signaling Technology (Danvers, MA, USA). Anti-CD206 and anti-CD86 were from BD Biosciences (San Diego, CA, USA). DHE (dihydroethidium), lipopolysaccharide (LPS), and NBT (4-nitro blue tetrazolium chloride) were purchased from Sigma-Aldrich Chemical Co. (St. Louis, MO, USA). The IL-1*β* and TNF*α* enzyme-linked immune sorbent assay (ELISA) kits were from R&D systems (Minneapolis, MN, USA). p22^phox^ and keap1 siRNA and control siRNA were purchased from Gene Pharma (Shanghai, China). The following sequences were used: p22^phox^-mus-91, sense 5′-GGCCAUGUGGGCCA ACGAATT-3′, antisense 5′-UUCGUUGGCCCACAUGGCCTT-3′; p22^phox^-mus-119, sense 5′-GCGUCUGGCCUGAUUCUCATT-3′, antisense 5′-UGAGAAUCAG GCCAGACGCTT-3′; p22^phox^-mus-465, sense 5′-GGACUCCCAUUGAGCCUA ATT-3′, antisense 5′-UUAGGCUCAAUGGGAGUCCTT-3′; keap1-mus-1927, sense 5′-CUCCAGCCCAGUCUUUAAATT-3′, antisense 5′-UUUAAAGACUGG GCUGGAGTT-3′; keap1-mus-3076, sense 5′-CUCCGCAGAAUGUUACU AUTT-3′, antisense 5′-AUAGUAACAUUCUGCGGAGTT-3′; keap1-mus-3463, sense 5′-GAAGCAAAUUGAUCAACAATT-3′, antisense 5′-UUGUUGAUCAAUU UGCUUCTT-3′; BAP31siRNA, sense 5′UUCUCCGAACGUGUCACGUTT-3′, antisense 5′-ACGUGACACGUUCGGA GAATT-3′; siRNA-BAP31, sense 5′-CCU CUACGCAGAGGUCUTT-3′, antisense 5′-GGAGATGCGTCTCCAGAAA-3′; sense 5′-CCAUGGAGCACUUCCACATT-3′, antisense 5′-GGTACCTCGTGAAGGTGTAA-3′; sense 5′-GGCUAUGCAGAAGCAGUCTT-3′, antisense 5′-CCG ATACGTCTTCGTCAGAA-3′; and control siRNA, sense 5′-UUCUCCG AACGUGUCACGUTT-3′, antisense 5′-ACGUGACACGUUCGGAGAATT-3′.

### 2.2. *In Vitro* Experiments

#### 2.2.1. Cell Culture

BV2 microglial cells and human neuroblastoma (SHSY5Y) cells were cultured in DMEM/F12 media containing 10% heat-inactivated FBS, 100 U/ml penicillin, and 100 *μ*g/ml streptomycin at 37°C in a 5% CO_2_-humidified incubator. The stable cell line (scramble and shBAP31 BV2 microglial cells) were cultured in the above complete DMEM/F12 with 0.4 *μ*g/ml blasticidin (BSD).

#### 2.2.2. Primary Microglia Cell Culture

Primary microglial cells were prepared from 1- to 3-day-old newborn pups (BAP31^fl/fl^ and LysM-Cre-BAP31^fl/fl^ mice) of either sex as described [16]. Briefly, brains were dissected, and the meninges were removed. Then, the cortices were digested using trypsin and through a 70 *μ*m cell strainer; then, cell suspensions were incubated in 25 cm^2^ flasks pretreated with poly-L-lysine. After 4-7 days, astrocytes were recovered and microglia were generated by the addition of DMEM medium containing 25% of L929 conditioned medium. Three to four days later, the confluent mixed glia cells were subjected to shaking at 37°C at 100 rpm for an hour. Microglial cells were resuspended in DMEM/F12 medium containing 25% L929 conditioned medium for experiments. The microglial cells were incubated with LPS (100 ng/ml) for 12 h after having been pretreated with or without apocynin (1 mM) [17] for 1 h, and then superoxide anion production was measured by dying with NBT and DHE.

#### 2.2.3. BAP31 shRNA and Transfection

The pL/shRNA/green fluorescent protein- (GFP-) mouse-BAP31 (shBAP31) lentiviral construct and control construct pL/shRNA/GFP (Scramble) were purchased from Novobio Technology (Shanghai, China). The following sequences were used: sense 5′-CACCG∗CCATGGCTTATAGATCATTATCGAAATAATGATCTATAAG CCATGG-3′ and antisense 5′-AAAACCATGGCTTATAGATCATTATTTCGATA AGATCTATAAGCCATGGC∗-3′. BV2 cells were infected with shBAP31 or scramble lentiviral construct (multiplicity of infection = 250) at 37°C for 72 h, then selected using 0.4 *μ*g/ml blasticidin to screen single cell clone and expansion in culture (containing 0.4 *μ*g/ml blasticidin) for 4 weeks. The knockdown efficiency was measured using Western blotting and RT-PCR when used for study.

#### 2.2.4. Measurement of O_2_^•-^ and H_2_O_2_ Production

*(1) Nitroblue Tetrazolium (NBT) Reduction Assay*. The NBT assay is simple, sensitive, and quantitative and can be used to determine the amounts of intracellular O_2_^•-^ produced by a wide variety of cells [18]. The quantitation of O_2_^•-^ production in BV2 cells and primary microglia cells was performed using a nitroblue tetrazolium reduction assay. Cells were seeded at a density of 2 × 10^4^ cells/well in 12-well culture plates for 24 h. Cells were treated with LPS (100 ng/ml) for 12 h; then, cells were incubated with nitroblue tetrazolium chloride for 2 h at 37°C. Cells were then washed with PBS and fixed in 2% paraformaldehyde (PFA). Images of cells were taken, and then reduced formazan particles, contained inside cells, were dissolved with 2 M KOH in DMSO and the absorbance was read at 630 nm on a multimode microplate reader (Bio-Tek, USA).

*(2) Measurement of H_2_O_2_*. The levels of H_2_O_2_ were measured by using a H_2_O_2_ assay kit from Beyotime Biotechnology. Briefly, scramble and shBAP31 cells were treated with LPS for 12 h; then, cells were washed with cold PBS and lysed using the assay buffer provided by the kit. After homogenization, the cell samples were centrifuged at 12000 × g for 5 min and the supernatant was collected. After deproteinization, the working solution containing an OxiRed probe and horseradish peroxidase (HRP) was added to samples according to the manufacturer's instructions. In the presence of HRP, the OxiRed probe reacts with H_2_O_2_ to produce a product with color (*λ*_max_ = 570 nm). The absorbance at 570 nm was read with a multimode microplate reader (Bio-Tek, USA). The amount of H_2_O_2_ was calculated according to the standard curve (*y* = 0.0069*x* + 0.064, *R*^2^ = 0.9985) and expressed as fold of scramble control cells.

*(3) DHE for Superoxide Anion Assay*. A dihydroethidium (DHE) fluorescent probe (Beyotime Biotechnology) was used for detecting O_2_^•-^ in the microglial cells, which were dehydrated and showed red signals [19]. Briefly, scramble and shBAP31 cells were treated with LPS for 12 h and incubated with 5 *μ*M DHE for 30 min at 37°C according to the manufacturer's instructions. After incubation, the cells were washed with PBS and the images of cells were taken; the images of intracellular accumulation of superoxide anion were taken by a Leica (Wetzlar, HE, Germany) scanning confocal microscope, and the red fluorescence intensity was quantified by using NIH ImageJ software and averaged for all groups.

#### 2.2.5. Determination of Lipid Peroxidation in Microglia

MDA, a marker of lipid peroxidation, was assessed using the thiobarbituric acid (TBA) method using a kit from Beyotime Biotechnology according to the manufacturer's instructions. MDA concentrations were then calculated by the absorbance of TBA reactive substances at 532 nm. Briefly, scramble and shBAP31 microglial cells were treated with LPS for 24 h, and the cultures were washed with ice-cold PBS, pooled and lysed with RIPA lysis buffer for 1 h at 37°C, then centrifuged (12000 × g at 4°C for 10 min). 200 *μ*l TBA was added to 100 *μ*l cellular lysate, and the mixture was boiled at 100°C for 15 min. After cooling, the mixture was centrifuged at 1000 × g for 10 min. MDA formation was measured at 532 nm, and results were expressed as nmol of MDA/mg of protein.

#### 2.2.6. Quantitative Real-Time PCR

Total RNA was isolated using the TRIzol Reagent (Carlsbad, CA, USA), and reverse transcription was performed using GoScript™ Reverse Transcription System (Promega, Madison, USA) according to the manufacturer's instructions. Quantitative real-time PCR (qRT-PCR) was performed using GoTaq® qPCR Master (Promega, Madison, USA) with a CFX96 Touch™ Real-Time PCR Detection System (Bio-Rad Laboratories, CA, USA). The experiments were repeated three times with three replicates per detection. The relative fold changes in the expression of each messenger RNA (mRNA) was calculated using the ΔΔCt method relative to the expression of GAPDH according to the following formula: 2^−ΔΔCt^(ΔΔCt = [(Ct_target gene_ − Ct_GAPDH_)_sample_ − (Ct_target gene_ − Ct_GAPDH_)_control_]) [20]. PCR primers for p22^phox^ (*Cyba*), p40^phox^ (*Ncf4*), p47^phox^ (*Ncf1*), p67^phox^ (*Ncf2*), gp91^phox^ (*Cybb*), IL-1*β*, TNF*α*, CD80, CD86, Arg1, CD206, Fizz1, Ym1, and GAPDH were designed as follows: p22^phox^ (*Cyba*) primer, sense 5′-GTGTGCTCATCTGTCTGCTG-3′, antisense 5′-TGGGTTTAGGCTCAATGGGA-3′; p40^phox^ (*Ncf4*) primer, sense 5′-TCTCATCTACCGCCGCTATC-3′, antisense 5′-CCATGTAGACTTTGGCTGGC-3′; p47^phox^ (*Ncf1*) primer, sense 5′-TGCCAGA TGAAGACAAAGCG-3′, antisense 5′-TTCACCTGCGTAGTTGGGAT-3′; p67^phox^ (*Ncf2*) primer, sense 5′-ACCGCGTATTGTTTGGCTTT-3′, antisense 5′-AGCCCCTTCT GTCCATTGAA-3′; gp91^phox^ (*Cybb*) primer, sense 5′-GGTTTTGGCGATCTCAGC AA-3′, antisense 5′-ACTGTCCCACCTCCATCTTG-3′; IL-1*β* primer, sense 5′-TGACGGACCCCAAAAGATGA-3′, antisense 5′-TCTCCACAGCCACAATGA GT-3′; TNF*α* primer, sense 5′-CCCTCACACTCAGATCATCTTCT-3′, antisense 5′-GCTACGACGTGGGCTACAG-3′; CD80 primer, sense 5′-TGGCCCGAGTATAAGA ACCG-3′, antisense 5′-CCGGAAGCAAAGCAGGTAAT-3′; CD86 primer, sense 5′-GCACGTCTAAGCAAGGTCAC-3′, antisense 5′-CATATGCCACACACCATCCG-3′; Arg1 primer, sense 5′-GTCGGGAAGGAAGAAAAGGC-3′, antisense 5′-TGCCGT GTTCACAGTACTCT-3′; CD206 primer, sense 5′-TGGATGGATGGGAGCAAAGT-3′, antisense 5′-GCTGCTGTTATGTCTCTGGC-3′; Fizz1 primer, sense 5′-GAACTTCT TGCCAATCCAGCT-3′, antisense 5′-CTCCCAAGATCCACAGGCAA-3′; and Ym1 primer, sense 5′-GGGTAATGAGTGGGTTGGT-3′, antisense 5′-CCACGGCACCTCCTAA ATTG-3′.

#### 2.2.7. Western Blot Analysis

Cells were collected and lysed with RIPA lysis buffer (1 mol/l Tris-HCl, pH 7.4; 1% Triton X-100; 1% sodium deoxycholate; and 150 mM NaCl 0.1% SDS) with protease and phosphatase inhibitor. The lysates were centrifuged at 12000 × g for 15 min at 4°C to produce whole-cell extracts. Protein concentrations were measured by the micro-BCA protein assay kit (Thermo Fisher Scientific, Waltham, MA, USA). The same amount of total protein lysates was separated by 12% SDS-PAGE and transferred to Immobilon polyvinylidene difluoride (PVDF) membranes (Millipore). After blocking with 5% nonfat milk in PBS, immunoblots were incubated with primary antibodies overnight at 4°C, followed by treatment with HRP-linked secondary antibodies. The intensity of immune-reactive bands was quantified using Image Lab Software.

#### 2.2.8. Flow Cytometry Analysis

Scramble and shBAP31 microglial cells were treated with LPS (100 ng/ml) for 24 h, and the cells were harvested; then, 1 × 10^6^ cells were fixed with 2% PFA for 30 min, punched with saponin for 20 min, and then blocked with BSA for 1 h. Subsequently, cells were suspended in 100 *μ*l primary antibody (CD86 and CD206; 1 : 1000 in BSA) for 50 min on ice. Flow cytometry analyses were performed using BD Accuri™ C6 Flow Cytometer.

#### 2.2.9. Enzyme-Linked Immunosorbent Assay

The levels of interleukin-1*β* (IL-1*β*) and TNF*α* in the culture supernatants were measured by ELISA kits according to the manufacturer's instructions (R&D Systems, Minneapolis, MN). Briefly, scramble and shBAP31 microglial cells were treated with LPS (100 ng/ml) for 24 h and culture supernatants were harvested; the medium was collected and centrifuged for 20 min at 1000 × g to remove the pellet. A volume of each 20 *μ*l of the supernatant was sampled for measuring IL-1*β* and TNF*α* according to the manufacturer's protocol.

#### 2.2.10. Nitrite Assay

Accumulation of nitrite (NO^2-^) in culture supernatant fluids was measured by the Griess assay. Microglial cells (5 × 10^4^ cells/well) were plated into 96-well plates, then treated with LPS (100 ng/ml) for 24 h. Then, 50 *μ*l culture supernatant fluids were mixed with 50 *μ*l Griess reagent at 37°C. Fifteen minutes later, the absorbance was determined at 540 nm using a multimode microplate reader. Quantifications of NO^2-^ after LPS treatment were calculated as fold of untreated scramble cells.

#### 2.2.11. BV2 Microglia Conditioned Medium-Induced SHSY5Y Neurotoxicity

The effect of microglia conditioned medium (MCM) on the viability of SHSY5Y cells was measured using the MTT assay [21]. Scramble and shBAP31 cells were stimulated with LPS (100 ng/ml) for 24 h. Stimulation was terminated by collecting conditioned medium from the cells and centrifuged and stored at -80°C. SHSY5Y neurons cells were seeded at a density of 2 × 10^5^ cells/ml in 96-well cell culture plates and incubated at 37°C. When cells reach confluence, the culture medium was removed and replaced with 100 *μ*l of conditioned medium and DMEM medium containing LPS and/or apocynin and further incubated for 48 h. Stimulation was terminated by adding 20 *μ*l of MTT (5 mg/ml in PBS) solution and incubating for 4 h at 37°C; then, the supernatant was removed, and 150 *μ*l dimethyl sulfoxide (DMSO) was added to solubilize the formazan crystals. The absorbance was measured at 490 nm using a multimode microplate reader.

### 2.3. *In Vivo* Experiments

#### 2.3.1. Animal and Surgical Procedures

Mice with BAP31 deficiency in microglia were generated by crossing transgenic mice expressing Cre-recombinase under the lysozyme M (LysM) promoter with mice carrying a BAP31 gene flanked by LoxP sites [16]. The mice (BAP31^fl/fl^ and LysM-Cre-BAP31^fl/fl^ mice) were injected intraperitoneally (i.p.) with LPS (250 *μ*g/kg/day) or saline for 1 week (8 mice per group) to make a model of oxidative stress in the brain according to a previous study [22–24]. Apocynin (30 mg/kg) was orally administrated once daily before LPS injection [25, 26] to verify BAP31 functions through NADPH oxidase. The mice were divided into six groups: BAP31^fl/fl^; BAP31^fl/fl^ + LPS; LysM-Cre-BAP31^fl/fl^; LysM-Cre-BAP31^fl/fl^ + LPS; LysM-Cre-BAP31^fl/fl^ + apo; and LysM-Cre-BAP31^fl/fl^ + LPS + apo for the study. Mice were sacrificed, and brains were used for superoxide anion and morphological analysis. All animal experiments were carried out as per the National Institutes of Health Guide for the Care and Use of Laboratory Animals, as well as according to the guidelines of animal handling of our university authority.

#### 2.3.2. Measurement of Reactive Oxygen Species Levels in Brain

To assess superoxide anion production in the brain, the mice (BAP31^fl/fl^ and LysM-Cre-BAP31^fl/fl^ mice) were deeply anesthetized and perfused with saline and 4% paraformaldehyde; the brains were fixed in 4% paraformaldehyde in PBS overnight at 4°C; and the brains were first dehydrated by placing the tissue for 30 min in each of 70%, 95%, and 100% ethanol solutions, and then embedded (*n* = 8 per group for each experiment). The brains were cut at 10 *μ*m thickness using a microtome blade (Leica, Wetzlar, HE, Germany), and sections (10 *μ*m) were incubated with 5 *μ*mol/l fluorescent dye dihydroethidium (DHE, Molecular Probes) at 37°C for 30 min in a humidified chamber and protected from light. Digital images were captured by a Leica (Wetzlar, HE, Germany) scanning confocal microscope, and the red fluorescence intensity was quantified by using NIH ImageJ software. The fluorescence intensity was expressed relative to that of BAP31^fl/fl^.

#### 2.3.3. Immunofluorescence Staining

Brain sections from all groups were prepared as described in Section 2.3.2; then, endogenous peroxidase activity was blocked with 0.3% H_2_O_2_ for 10 min, washed in PBS, blocked with 5% BSA for 1 h, then species were incubated with the primary antibody (mouse 8-OHdG 1 : 200 in 10% goat serum PBS buffer) overnight at 4°C. The slices were then incubated with red fluorescent-conjugated secondary antibody (Thermo Fisher Scientific Inc.) for 1 h. After washing, the slices were incubated with rabbit anti-NeuN (1 : 1000 in 10% goat serum PBS buffer) followed by a green fluorescent-conjugated secondary antibody (Thermo Fisher Scientific Inc.) for another 1 h. Sections were then washed in PBS, and DAPI was used as the blue nuclear stain. Sections were viewed and processed in a Leica scanning confocal microscope. NeuN-positive cells were counted using ImageJ software (NIH) with a DAPI counterstain. The relative intensity of fluorescence in NeuN-positive cells/field of view was used for statistical analysis.

### 2.4. Statistical Analysis

**Figure 1:**
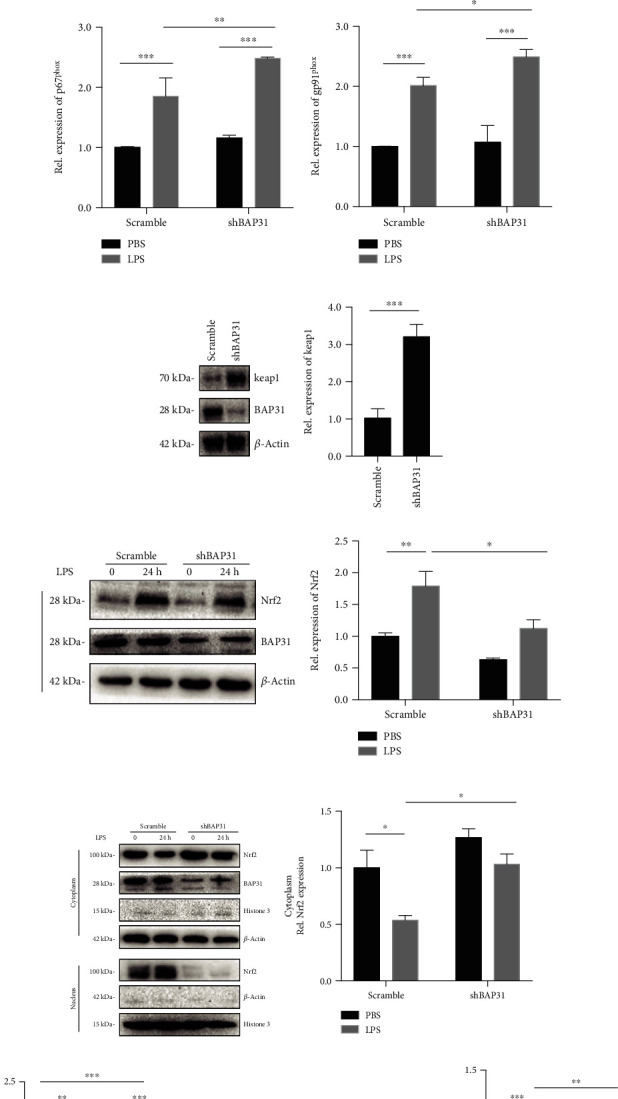
BAP31 deficiency upregulates p22^phox^ and keap1 and inhibits Nrf2/HO-1 signaling in LPS-treated BV2 microglia. Scramble and shBAP31 were treated with LPS (100 ng/ml) for 24 h. The mRNA levels of p22^phox^ (a), p40^phox^ (b), p67^phox^ (c), and gp91^phox^ (d) were analyzed with RT-PCR. (e) Representative Western blots showing the expression levels of keap1 in scramble and shBAP31 BV2 cells. (f) Scramble and shBAP31 cells were treated with LPS for 24 h. The protein levels of Nrf2, BAP31, and *β*-actin were analyzed by Western blotting. (g) Scramble and shBAP31 BV2 cells were treated with LPS for 24 h; the cytosolic and nuclear fractions of Nrf2 were analyzed by Western blotting with antibodies against Nrf2, histone, and *β*-actin. (h) Immunoblots for Nrf2 in cytosolic fractions were quantified and normalized to *β*-actin protein. (i) Immunoblots for Nrf2 in nuclear fractions were quantified and normalized to histone protein. (j) Representative Western blots showing the expression levels of HO-1 in scramble and shBAP31 BV2 cells after LPS treatment for 24 h. All the data are indicated as mean ± SEM of three independent experiments. ^∗^*p* < 0.05, ^∗∗^*p* < 0.01, and ^∗∗∗^*p* < 0.001 versus the control group.

Statistical analyses were conducted with GraphPad Prism 7.0 Software (GraphPad, La Jolla, CA, USA) according to previous studies [27, 28]. Data are expressed as the mean ± SEM. Student's *t*-test was used to compare two groups and was always used as two-tailed in Figure 1(e). For other Figures, analyses were performed using two-way ANOVA followed by Tukey's multiple-comparison analyses, *p* values < 0.05 were considered significant. ^∗^*p* < 0.05, ^∗∗^*p* < 0.01, and ^∗∗∗^*p* < 0.001, and ns (no significant difference) denotes the significance thresholds.

## 3. Results

### 3.1. BAP31 Deficiency Exacerbates LPS-Induced Superoxide Anion Generation in Microglia

**Figure 2:**
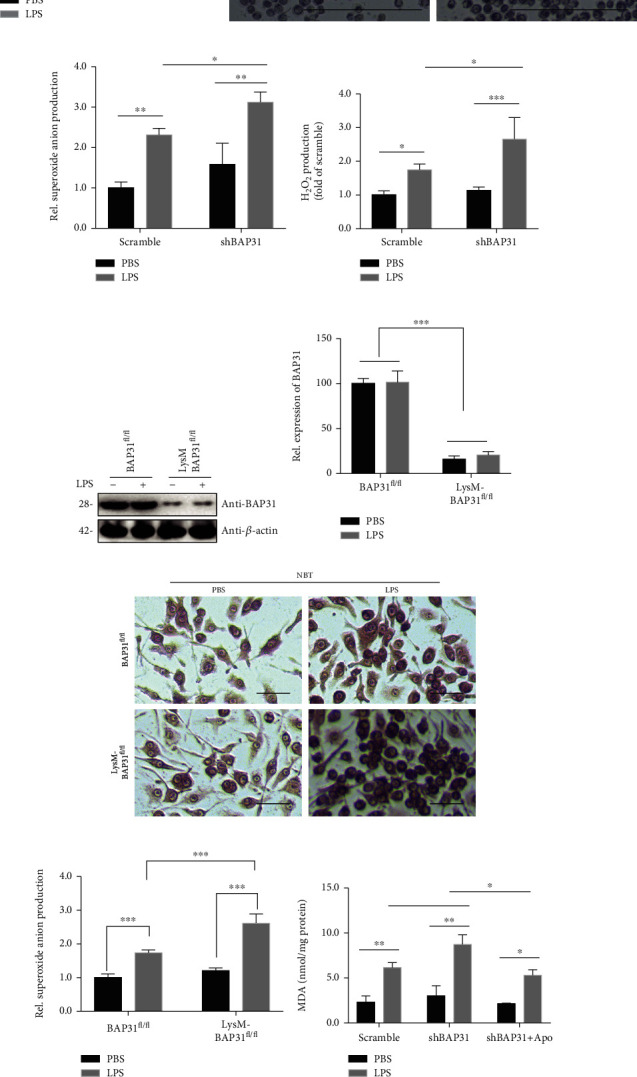
BAP31 deficiency exacerbates LPS-induced superoxide anion generation in microglia. (a) The protein level of BAP31 in scramble and shBAP31 BV2 microglial cells. (b) Scramble and shBAP31 cells were stimulated with LPS (100 ng/ml) for 12 h. Superoxide anion were measured by staining with NBT and observed by confocal microscopy. Scale bars = 100 *μ*m. (c) Intracellular superoxide anion detected by NBT staining in (b) was quantified using a multimode microplate reader. (d) Scramble and shBAP31 cells were stimulated with LPS (100 ng/ml) for 12 h; H_2_O_2_ production was measured using a multimode microplate reader. (e, f) The protein level of BAP31 in primary microglial cells from BAP31^fl/fl^ and LysM-Cre-BAP31^fl/fl^ mice. (g) Visualization of NBT staining in primary microglial cells from BAP31^fl/fl^ and LysM-Cre-BAP31^fl/fl^ mice after LPS treatment (100 ng/ml) for 12 h. (h) Intracellular superoxide anion detected by NBT staining in (g) were quantified using a multimode microplate reader. Scale bars = 10 *μ*m. (i) Scramble and shBAP31 cells were pretreated with apocynin for 1 h and then stimulated with LPS (100 ng/ml) for 24 h. Lipid peroxidation (MDA) levels were measured using a multimode microplate reader. Data are indicated as the mean ± SEM of three independent experiments. ^∗^*p* < 0.05, ^∗∗^*p* < 0.01, and ^∗∗∗^*p* < 0.001 versus the control group.

LPS-induced superoxide anion production was successfully established in earlier studies [29]. First, we employed an shRNA approach to specifically knockdown BAP31 in microglial BV2 cells, and the protein level of BAP31 was reduced by 80% compared with the scramble shRNA cells (Figure 2(a)). Experiments were then conducted to determine whether superoxide anion production was influenced by BAP31. As shown in Figures 2(b)–2(d), BAP31 deficiency showed an increasing tendency on the superoxide anion production. After stimulation with LPS, intracellular superoxide anion production was significantly upregulated in shBAP31 cells compared with scramble cells treated with LPS. In the NBT assay, superoxide anion production increased from 2.31 ± 0.09-fold to 3.11 ± 0.14-fold (Figures 2(b) and 2(c)), *F*_(1, 8)_ = 65.87, *p* < 0.001; DHE staining showed that intracellular superoxide anion significantly increased from 2.34 ± 0.10-fold to 3.28 ± 0.30-fold (supplementary Figure 1a and b), *F*_(1, 8)_ = 91.57, *p* < 0.001; and intracellular H_2_O_2_ significantly increased from 1.75 ± 0.18-fold to 2.65 ± 0.32-fold (Figure 2(d)), *F*_(1, 12)_ = 45.17, *p* < 0.001.

To further confirm the effect of BAP31 on superoxide anion production, primary microglial cells were used for superoxide anion production. As we reported earlier, BAP31 expression was blunted in microglia isolated from LysM-Cre-BAP31^fl/fl^ mice compared with BAP31^fl/fl^ mice (Figures 2(e) and 2(f)). Then, NBT assay results also indicated that BAP31 deficiency had no significant influence on superoxide production but increased LPS-induced superoxide production from 1.73 ± 0.04-fold to 2.61 ± 0.15-fold, *F*_(1, 8)_ = 147.7, *p* < 0.001 (Figures 2(g) and 2(h)).

A high quantity of superoxide and H_2_O_2_ has been reported to inflict direct damage to lipids, and MDA is a lipid damage marker. We next investigated the effect of BAP31 on LPS-induced MDA expression, and the results demonstrated that BAP31 deficiency had no effect on MDA expression but significantly increased LPS-induced MDA production compared with scramble cells treated with LPS (Figure 2(i)) from 6.12 ± 0.31 nmol/mg to 8.70 ± 0.60 nmol/mg. Apocynin significantly inhibited BAP31-deficiency-induced MDA expression from 8.70 ± 0.60 nmol/mg to 5.26 ± 0.35 nmol/mg, *F*_(1, 12)_ = 143.4, *p* < 0.001.

### 3.2. BAP31 Deficiency Enhanced the P22^phox^ Protein Level but Inhibited the Nrf2/HO-1 Signaling Pathways in Microglia

An increasing body of research suggests that superoxide anion production is mainly regulated by NADPH oxidase [30]. We detected whether BAP31 could regulate superoxide anion production by NADPH oxidase. Scramble and shBAP31 microglial cells were stimulated with LPS for 24 h, and then the RT-PCR assay was used to detect the mRNA levels of NOX subunits, including p22^phox^, p40^phox^, p47^phox^, p67^phox^, and gp91^phox^. As illustrated in Figures 1(a)–1(d), BAP31 deficiency significantly increased the mRNA level of p22^phox^ from 1.00 ± 0.02-fold to 1.91 ± 0.05-fold, *F*_(1, 8)_ = 700.8, *p* < 0.001, but the other subunits showed no significant change. After LPS treatment, all the subunits were increased in both scramble and shBAP31 cells except p47^phox^ (supplementary Figure 2), but BAP31 deficiency significantly increased the mRNA levels of NOX subunits compared with scramble cells treated with LPS (p22^phox^ increased from 2.05 ± 0.04-fold to 3.64 ± 0.08-fold, *F*_(1, 8)_ = 570.8, *p* < 0.001; p40^phox^ from 1.84 ± 0.10-fold to 2.38 ± 0.03-fold, *F*_(1, 8)_ = 267.4, *p* < 0.001; p67^phox^ from 1.87 ± 0.17-fold to 2.49 ± 0.01-fold, *F*_(1, 8)_ = 150.8, *p* < 0.001; gp91^phox^ from 1.81 ± 0.13-fold to 2.48 ± 0.05-fold, *F*_(1, 8)_ = 176.40, *p* < 0.001).

To investigate whether the keap1/Nrf2 signaling pathway was regulated by BAP31, we first detected whether the protein levels of keap1 and Nrf2 were influenced by BAP31. The results showed that the protein level of keap1 was significantly increased from 1.02 ± 0.15-fold to 3.21 ± 0.19-fold, *p* < 0.001, whereas the Nrf2 protein was not influenced by the deficiency of BAP31; after LPS stimulation, the protein level increased, but BAP31-deficient cells had a low level of Nrf2 compared with scramble cells (Figures 1(e) and 1(f)), *F*_(1, 12)_ = 21.85, *p* < 0.001. Then, we assessed the translocation of Nrf2; scramble and shBAP31 cells were treated with LPS for 24 h, after which the nuclei were separated from the cytoplasm. As shown in Figures 1(g)–1(i), upon LPS stimulation, translocation of Nrf2 from the cytoplasm to the nucleus significantly increased in scramble cells, but BAP31-deficient cells showed an inhibited translocation of Nrf2 compared with scramble cells. In the cytoplasm, the protein level of Nrf2 decreased from 1.00 ± 0.16-fold to 0.54 ± 0.04-fold in scramble cells, *F*_(1, 8)_ = 14.65, *p* = 0.008, while in shBAP31 cells, the protein level of Nrf2 decreased from 1.27 ± 0.08-fold to 1.03 ± 0.09-fold, *F*_(1, 8)_ = 14.65, *p* = 0.39; accordingly, in the nucleus, the protein level of Nrf2 increased from 1.00 ± 0.11-fold to 1.74 ± 0.12-fold in scramble cells, *F*_(1, 8)_ = 107.7, *p* < 0.01, while in shBAP31 cells, the protein level of Nrf2 increased from 0.25 ± 0.04-fold to 0.39 ± 0.12-fold. Next, we detected the expression of the antioxidant protein HO-1 in scramble and shBAP31 BV2 cells stimulated with LPS for 24 h. HO-1 protein levels were assessed by Western blotting. As shown in Figure 1(j), BAP31 deficiency significantly decreased LPS-induced HO-1 protein expression level from 1.00 ± 0.06-fold to 0.70 ± 0.04-fold compared with scramble cells after LPS treatment, *F*_(1, 8)_ = 118.7, *p* < 0.01.

### 3.3. BAP31 Deficiency Induces Inflammatory Cytokine Production in LPS-Stimulated Cells

**Figure 3:**
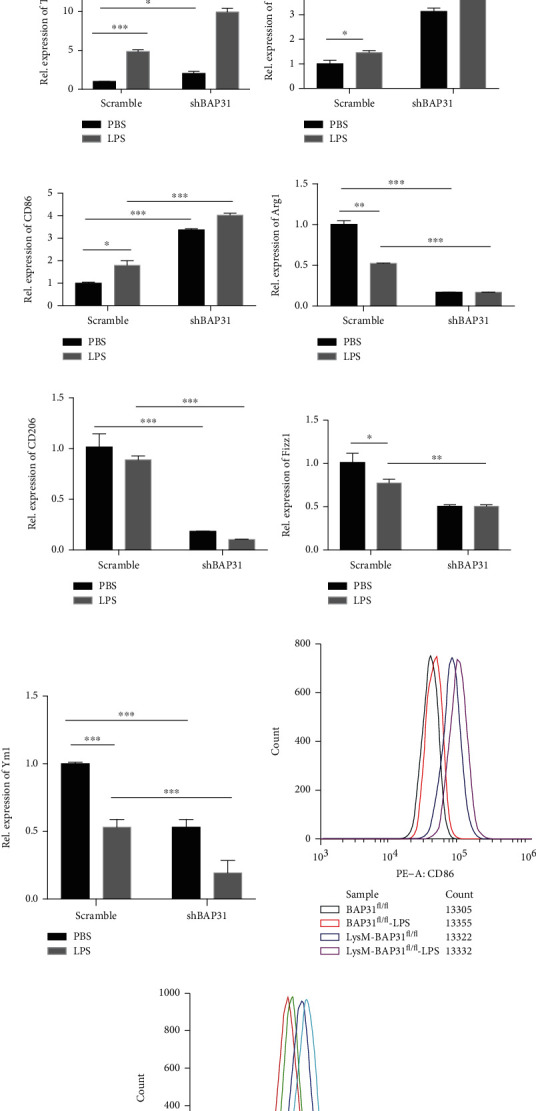
BAP31 deficiency induces proinflammatory cytokine production in LPS-stimulated cells. Scramble and shBAP31 BV2 cells were treated with LPS (100 ng/ml) for 24 h. The mRNA levels of BAP31 (a), IL-1*β* (b), TNF*α* (c), CD80 (d), CD86 (e), Arg1 (f), CD206 (g), Fizz1 (h), and Ym1 (i) were analyzed by RT-PCR. Flow cytometry shows that the expression of CD86 (j) and CD206 (k) in primary microglial cells were treated with LPS (100 ng/ml) for 24 h. All the data are indicated as mean ± SEM of three independent experiments. ^∗^*p* < 0.05, ^∗∗^*p* < 0.01, and ^∗∗∗^*p* < 0.001 versus the control group.

Reactive oxygen species are key signaling molecules that play an important role in the progression of inflammatory disorders [31]. We next investigated the effect of BAP31 on the mediators of microglial cells. IL-1*β*, TNF*α*, CD80, and CD86 were used as markers of proinflammatory mediators, while Arg1, CD206, Fizz1, and Ym1 were markers of anti-inflammatory mediators. BAP31 mRNA expression was significantly decreased compared with scramble cells (Figure 3(a)). As illustrated in Figure 3, we found that BAP31 deficiency in microglial cells increased IL-1*β*, TNF*α*, CD80, and CD86 mRNA expression: IL-1*β* increased from 1.01 ± 0.09-fold to 6.64 ± 0.21-fold (Figure 3(b)), *F*_(1, 8)_ = 285.5, *p* < 0.001; TNF*α* increased from 1.00 ± 0.01-fold to 2.04 ± 0.15-fold (Figure 3(c)), *F*_(1, 8)_ = 299.7, *p* < 0.001; CD80 increased from 1.01 ± 0.08-fold to 3.14 ± 0.08-fold (Figure 3(d)), *F*_(1, 8)_ = 3.87, *p* < 0.001; and CD86 increased from 1.00 ± 0.03-fold to 3.14 ± 0.08-fold (Figure 3(e)), *F*_(1, 8)_ = 1185, *p* < 0.001. It also decreased the levels of Arg1, CD206, Fizz1, and Ym1 mRNA. After LPS treatment, the gene expression of proinflammatory mediators in shBAP31 cells was significantly upregulated compared with scramble cells: IL-1*β* increased from 5.80 ± 0.26-fold to 41.63 ± 2.33-fold (Figure 3(b)), *F*_(1, 8)_ = 309.9, *p* < 0.001; TNF*α* from 4.97 ± 0.05-fold to 9.94 ± 0.29-fold (Figure 3(c)), *F*_(1, 8)_ = 497.8, *p* < 0.001; CD80 from 1.46 ± 0.05-fold to 4.20 ± 0.28-fold (Figure 3(d)), *F*_(1, 8)_ = 247.3, *p* < 0.001; and CD86 from 1.80 ± 0.12-fold to 4.03 ± 0.05-fold (Figure 3(e)), *F*_(1, 8)_ = 1.16, *p* < 0.001. However, anti-inflammatory mediators were significantly downregulated compared with scramble cells (Figures 3(f)–3(i)) after LPS treatment.

Then, we verified the above results by flow cytometry. The primary microglial cells from BAP31^fl/fl^ and LysM-Cre-BAP31^fl/fl^ mice were specifically stained with CD86 and CD206, respectively. As shown in Figure 3(j), BAP31 deficiency increased the percentage of CD86-positive cells; after LPS stimulation, CD86-positive cells were significantly upregulated. As shown in Figure 3(k), CD206-positive cells were downregulated in BAP31-deficient cells; after LPS stimulation, CD206-positive cells were reduced further, which was consistent with the RT-PCR results.

### 3.4. Knockdown of p22^phox^ or Keap1 Alleviates LPS-Induced Superoxide Anion Production

**Figure 4:**
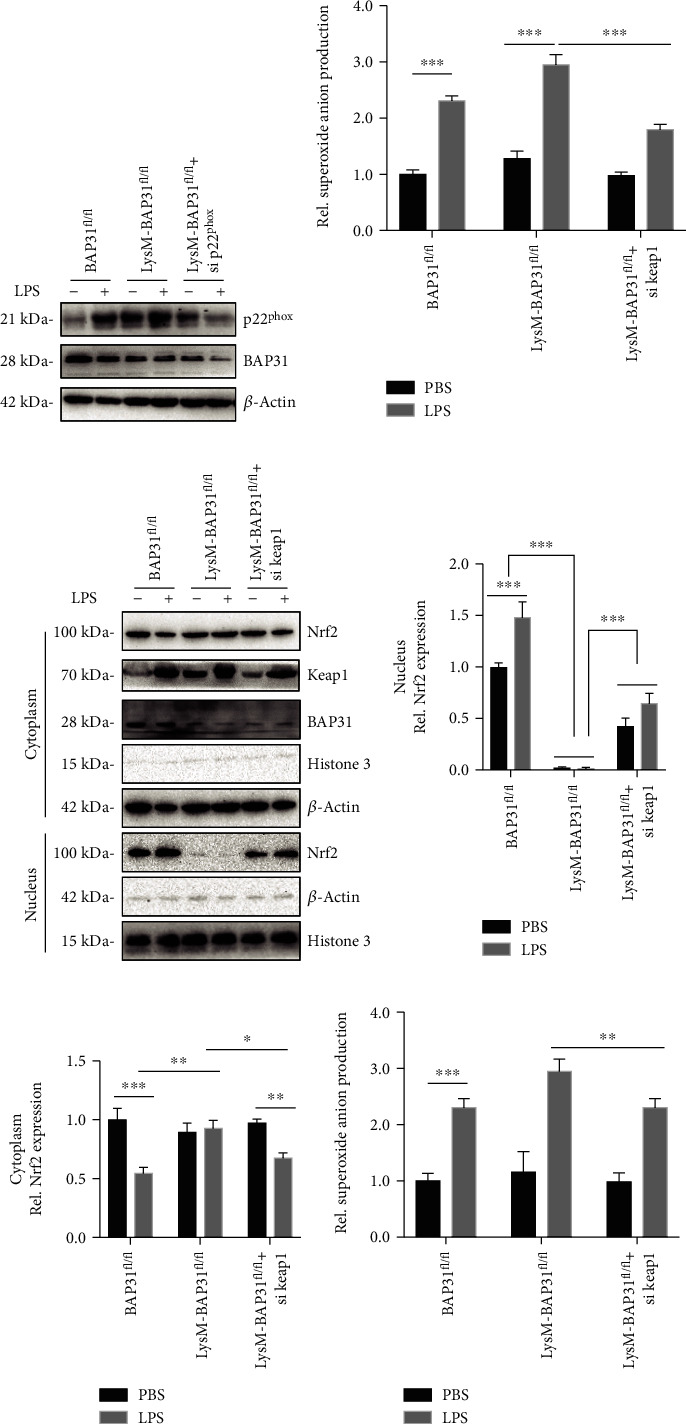
Knockdown of p22^phox^ or keap1 prevents BAP31-deficiency-induced superoxide anion production upon LPS stimulation. (a, b) Primary microglial cells were transfected with p22^phox^ siRNA for 60 h, followed by treatment with LPS for 12 h. The relative superoxide anion levels were measured by staining with NBT and quantified using a multimode microplate reader. (c, d, e) Primary microglial cells were transfected with keap1 siRNA for 60 h, followed by treatment with LPS for 24 h. The cytosolic and nuclear fractions were analyzed by Western blotting with antibodies against Nrf2, histone, and *β*-actin. (f) Primary microglial cells were transfected with keap1 siRNA for 60 h, followed by treatment with LPS for 12 h. The relative superoxide anion was measured by staining with NBT and quantified using a multimode microplate reader. All the data are indicated as mean ± SEM of three independent experiments. ^∗^*p* < 0.05, ^∗∗^*p* < 0.01, and ^∗∗∗^*p* < 0.001 versus the control group.

To explore the mechanism of BAP31 in superoxide anion production, we first knocked down p22^phox^ by siRNA to detect superoxide anion production in BAP31-deficient cells. Superoxide anion production was measured using the DHE assay. As shown in Figures 4(a) and 4(b), p22^phox^ deficiency decreased superoxide production from 2.95 ± 0.13-fold to 1.79 ± 0.04-fold, *F*_(2, 12)_ = 20.8, *p* < 0.01. Next, we examined whether keap1 silencing could reverse the inhibited translocation of Nrf2 induced by BAP31 deficiency. Primary BAP31-deficient microglial cells were transfected with keap1 siRNA for 60 h, the cells were exposed to LPS for 24 h, and then the nuclei were separated from the cytoplasm. As shown in Figures 4(c), 4(d), and 4(e), keap1 deficiency significantly reversed the translocation of Nrf2 compared with BAP31 deficiency, and the protein level of Nrf2 in the nucleus increased from 0.02 ± 0.07-fold to 0.64 ± 0.01-fold, *F*_(2, 12)_ = 317.1, *p* < 0.001. We then assessed whether BAP31-deficiency-induced superoxide anion production was alleviated by keap1 silencing. As shown in Figure 4(f), in the presence of keap1 deficiency, superoxide production decreased from 2.95 ± 0.13-fold to 2.31 ± 0.09-fold, *F*_(2, 12)_ = 7.06, *p* < 0.01.

### 3.5. Apocynin Prevents BAP31-Deficiency-Induced Superoxide Anion Production upon LPS Stimulation

**Figure 5:**
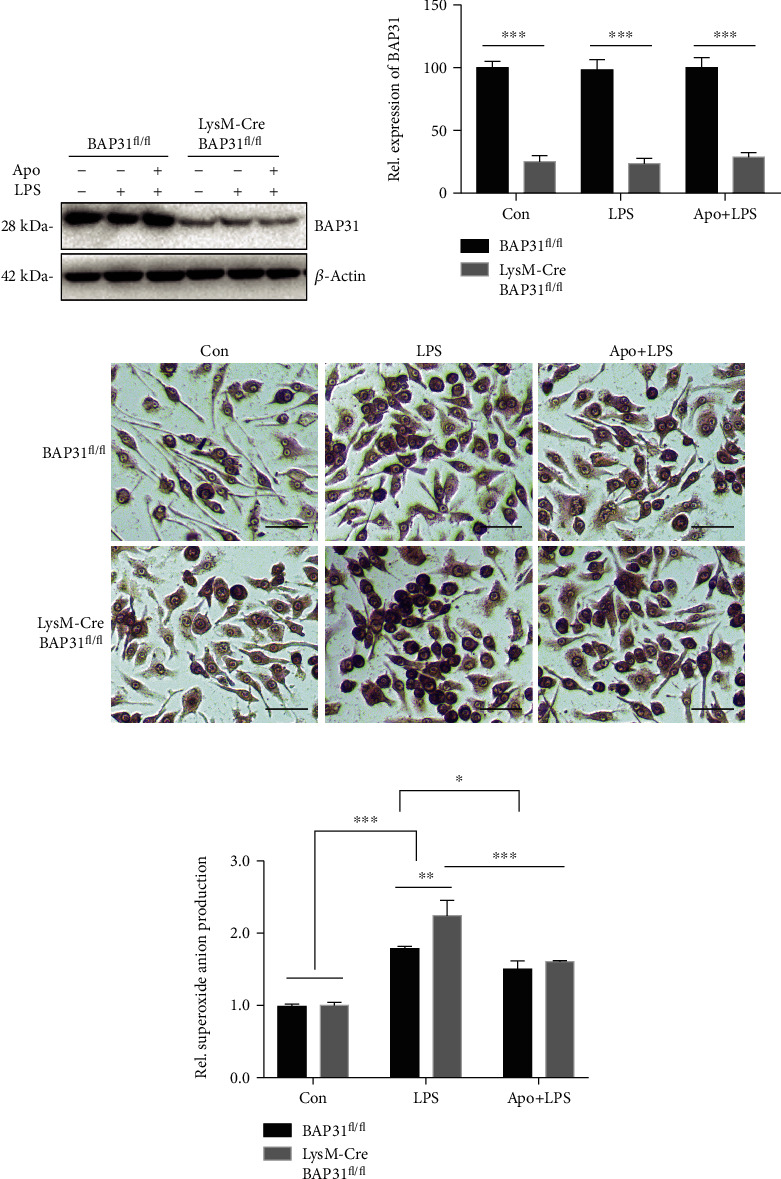
Apocynin alleviates superoxide anion production caused by the deficiency of BAP31. (a, b) Protein levels of BAP31 in primary microglial cells from BAP31^fl/fl^ and LysM-Cre-BAP31^fl/fl^ mice. (c, d) Visualization of NBT staining in primary microglial cells from BAP31^fl/fl^ and LysM-Cre-BAP31^fl/fl^ mice pretreated with apocynin for 1 h and then stimulated with LPS (100 ng/ml) for 12 h. Intracellular superoxide anion detected by NBT staining were quantified using a multimode microplate reader. Scale bars = 10 *μ*m. All the data are indicated as mean ± SEM of three independent experiments. ^∗^*p* < 0.05, ^∗∗^*p* < 0.01, and ^∗∗∗^*p* < 0.001 versus the control group.

To further clarify whether NADPH oxidase inhibitor could alleviate BAP31-deficiency-induced superoxide anion production, we used apocynin to assess superoxide anion production in BAP31-deficient microglial cells stimulated with LPS. The protein level of BAP31 is shown in Figures 5(a) and 5(b). Primary microglial cells were incubated with apocynin for 1 h and then stimulated with LPS for 24 h. The subunits of NOX were detected (supplementary Figure 3), and results showed that apocynin alleviated NOX subunits expression induced by BAP31 deficiency after LPS stimulation, decreasing p22^phox^ from 5.04 ± 0.29-fold to 3.39 ± 0.58-fold, *F*_(2, 12)_ = 35.83, *p* < 0.05; p40^phox^ from 1.57 ± 0.03-fold to 0.71 ± 0.07-fold, *F*_(2, 12)_ = 123.7, *p* < 0.001; p67^phox^ from 2.05 ± 0.13-fold to 1.78 ± 0.03-fold, *F*_(2, 12)_ = 30.92, *p* < 0.001; and gp91^phox^ from 1.84 ± 0.08-fold to 1.17 ± 0.07-fold, compared with cells from BAP31^fl/fl^ mice stimulated with LPS, *F*_(2, 12)_ = 41.4, *p* < 0.001.

Subsequently, we examined whether apocynin treatment influenced superoxide anion production in BAP31-deficient microglial cells. Primary microglial cells from BAP31^fl/fl^ and LysM-Cre-BAP31^fl/fl^ mice were treated with apocynin for 1 h, cells were stimulated with LPS, and then superoxide production was detected by the NBT and DHE assay. As shown in the NBT assay (Figures 5(c) and 5(d)), apocynin treatment decreased LPS-induced superoxide production caused by BAP31 deficiency from 2.24 ± 0.12-fold to 1.61 ± 0.01-fold, *F*_(2, 12)_ = 152.5, *p* < 0.001. As shown in the DHE assay (supplementary Figure 4), apocynin treatment decreased LPS-induced superoxide production caused by BAP31 deficiency from 3.98 ± 0.24-fold to 1.97 ± 0.07-fold, *F*_(2, 12)_ = 143.3, *p* < 0.001.

### 3.6. Knockdown of p22^phox^ or Keap1 Prevents Proinflammatory Cytokine Production in Microglia

**Figure 6:**
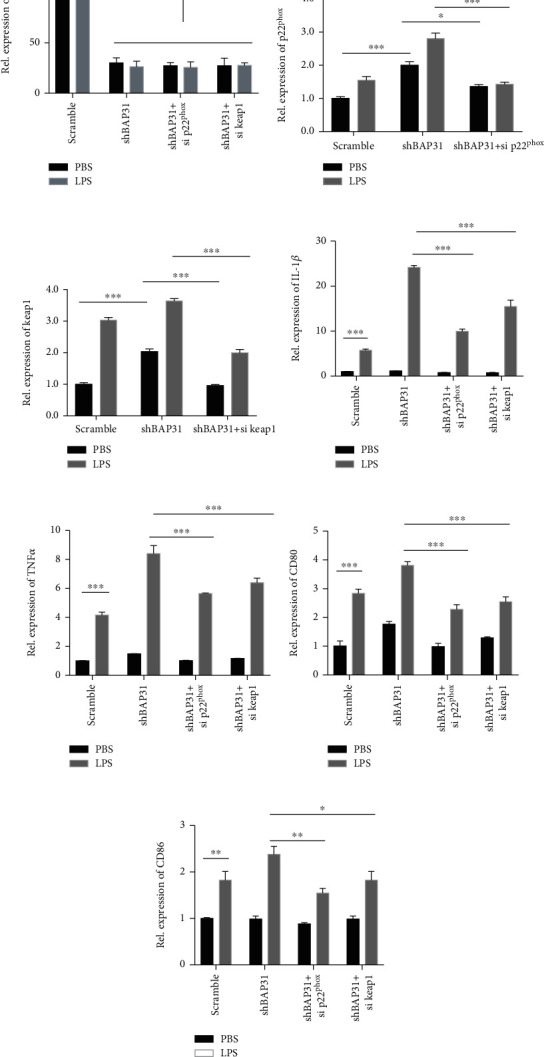
Knockdown of p22^phox^ or keap1 prevents proinflammatory cytokine production in BV2 microglia. Scramble and shBAP31 were transfected with p22^phox^ or keap1 siRNA for 60 h, followed by treatment with LPS for 24 h. The mRNA levels of BAP31 (a), p22^phox^ (b), keap1 (c), IL-1*β* (d), TNF*α* (e), CD80 (f), and CD86 (g) were analyzed by RT-PCR. All the data are indicated as mean ± SEM of three independent experiments. ^∗^*p* < 0.05, ^∗∗^*p* < 0.01, and ^∗∗∗^*p* < 0.001 versus the control group.

Next, we analyzed whether knockdown of p22^phox^ or keap1 could alleviate LPS-induced proinflammatory cytokine production in microglial cells with BAP31 deficiency. The shBAP31 cells were transfected with p22^phox^ or keap1 siRNA for 60 h and then stimulated with LPS for 24 h, and the mRNA levels of BAP31, p22^phox^, keap1, and inflammatory cytokines were assessed by RT-PCR. BAP31 was significantly decreased in shBAP31 cells (Figure 6(a)), and the mRNA level of p22^phox^ and keap1 were significantly decreased after siRNA treatment (Figures 6(b) and 6(c)). As shown in Figures 6(d)–6(g), when p22^phox^ was knocked down in BAP31-deficient cells, the gene expression of proinflammatory cytokines was significantly downregulated compared with shBAP31 cells after LPS treatment (IL-1*β* decreased from 24.14 ± 0.24-fold to 9.96 ± 0.28-fold, *F*_(3, 16)_ = 318.3, *p* < 0.001; TNF*α* decreased from 8.41 ± 0.55-fold to 5.64 ± 0.04-fold, *F*_(3, 16)_ = 35.14, *p* < 0.001; CD80 decreased from 3.81 ± 0.07-fold to 2.28 ± 0.10-fold, *F*_(3, 16)_ = 82.43, *p* < 0.001; and CD86 decreased from 2.38 ± 0.17-fold to 1.55 ± 0.10-fold, *F*_(3, 16)_ = 5.05, *p* < 0.01). When keap1 was knocked down in BAP31-deficient cells, the gene expression of proinflammatory cytokines was also significantly downregulated compared with shBAP31 cells after LPS treatment (IL-1*β* decreased from 24.14 ± 0.24-fold to 15.50 ± 0.24-fold, *F*_(3, 16)_ = 318.3, *p* < 0.001; TNF*α* decreased from 8.41 ± 0.55-fold to 6.40 ± 0.31-fold, *F*_(3, 16)_ = 35.14, *p* < 0.001; CD80 decreased from 3.81 ± 0.07-fold to 2.55 ± 0.11-fold, *F*_(3, 16)_ = 82.43, *p* < 0.001; and CD86 decreased from 2.38 ± 0.17-fold to 1.83 ± 0.19-fold, *F*_(3, 16)_ = 5.05, *p* < 0.05.

### 3.7. Apocynin Prevents Proinflammatory Cytokine Production in Microglia and Alleviates LPS-Induced Neurotoxicity among Cocultures

To further verify BAP31 regulation of cytokine production through superoxide anion, we used apocynin to assess cytokine production in BAP31-deficient microglial cells. Scramble and shBAP31 cells were stimulated with LPS for 24 h after having been pretreated with or without apocynin for 1 h, and then proinflammatory and anti-inflammatory cytokines were detected. As shown in supplementary Figure 5b-e, apocynin significantly inhibited the expression of proinflammatory cytokines in BAP31-deficient cells after LPS stimulation (IL-1*β* decreased from 21.27 ± 2.54-fold to 4.49 ± 0.17-fold, *F*_(2, 12)_ = 65.32, *p* < 0.001; TNF*α* decreased from 3.45 ± 0.03-fold to 2.45 ± 0.06-fold, *F*_(2, 12)_ = 233.9, *p* < 0.001; CD80 decreased from 10.07 ± 0.15-fold to 2.44 ± 0.84-fold, *F*_(2, 12)_ = 69.76, *p* < 0.001; CD86 decreased from 6.03 ± 0.05-fold to 2.45 ± 0.06-fold, *F*_(2, 12)_ = 643.6, *p* < 0.001; and apocynin significantly upregulated the expression of Arg1 from 0.05 ± 0.01-fold to 0.29 ± 0.02-fold (supplementary Figure 5f), *F*_(2, 12)_ = 93.87, *p* < 0.001; however, other anti-inflammatory cytokines showed no significant changes (supplementary Figures 5g-i).

**Figure 7:**
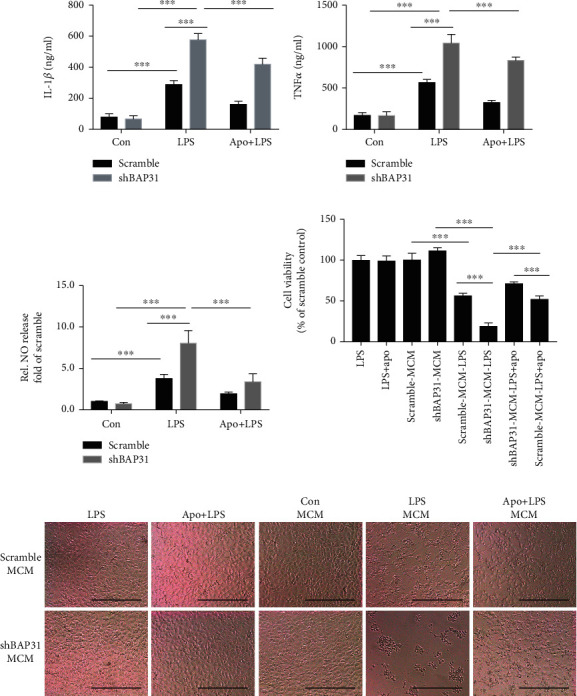
Apocynin prevents proinflammatory cytokine release in BV2 microglia and alleviates LPS-induced neurotoxicity among cocultures. (a, b, and c) Scramble and shBAP31 BV2 cells were treated with LPS (100 ng/ml) for 24 h, and the secreted protein levels of the cytokines IL-1*β* (a) and TNF*α* (b) in the supernatant were analyzed using ELISA kits. NO production (c) was measured by the Griess assay. (d, e) Visualization of SHSY5Y cells after coculture with microglial conditional medium (MCM) from scramble and shBAP31 BV2 cells exposed to LPS for 24 h after treatment with apocynin for 1 h. Cell viability was measured by the MTT assay. Scale bars = 200 *μ*m. All the data are indicated as mean ± SEM of three independent experiments. ^∗^*p* < 0.05, ^∗∗^*p* < 0.01, and ^∗∗∗^*p* < 0.001 versus the control group.

This inflammatory state is accompanied by increased cytokine production and has been shown to promote neuronal injury and death. To gain insight into the effect of BAP31-deficient microglia on neurons, we collected microglial conditioned medium (MCM) and detected the inflammatory cytokines IL-1*β*, TNF*α*, and NO release. As shown in Figure 7, BAP31 deficiency significantly increased LPS-induced inflammatory cytokine release: IL-1*β* increased from 288.5 ± 14.58 pg/ml to 577.3 ± 23.02 pg/ml (Figure 7(a)), *F*_(2, 30)_ = 233, *p* < 0.001; TNF*α* increased from 566.2 ± 21.15 pg/ml to 1044 ± 60.17 pg/ml (Figure 7(b)), *F*_(2, 30)_ = 208.9, *p* < 0.001; and NO increased from 3.77 ± 0.21-fold to 8.04 ± 0.63-fold (Figure 7(c)), *F*_(2, 30)_ = 130.8, *p* < 0.001. Apocynin treatment significantly inhibited the release of proinflammatory cytokines in BAP31-deficient cells: IL-1*β* decreased from 577.3 ± 23.02 pg/ml to 419.3 ± 22.16 pg/ml (Figure 7(a)), *F*_(2, 30)_ = 233, *p* < 0.001; TNF*α* decreased from 1044 ± 60.17 pg/ml to 833.6 ± 23.34 pg/ml (Figure 7(b)), *F*_(2, 30)_ = 208.9, *p* < 0.001; and NO decreased from 8.04 ± 0.63-fold to 4.27 ± 0.66-fold (Figure 7(c)). These results were consistent with the intracellular results.

To investigate whether BAP31 influenced the survival of neurons through proinflammatory cytokines, SHSY5Y cells were incubated with microglial conditioned medium from scramble and shBAP31 BV2 cells stimulated with LPS pretreated with or without apocynin. After 48 h of incubation, as shown in Figures 7(d) and 7(e), LPS or apocynin alone have no effect on the survival of neurons, but the survival rate of SHSY5Y cocultured with MCM from shBAP31 cells treated with LPS decreased from 56.01 ± 1.55% to 18.78 ± 1.89% compared with SHSY5Y cocultured with MCM from scramble cells treated with LPS, *F*_(7, 32)_ = 192.3, *p* < 0.001. MCM from shBAP31 cells pretreated with apocynin and LPS resulted in an increased survival rate of SHSY5Y from 18.78 ± 1.89% to 51.93 ± 1.90% compared with shBAP31 cells stimulated with LPS, *F*_(7, 32)_ = 192.3, *p* < 0.001.

### 3.8. BAP31-Deficiency Increased Superoxide Anion Production *In Vivo* and Caused Damage and Death in Hippocampus Neurons

**Figure 8:**
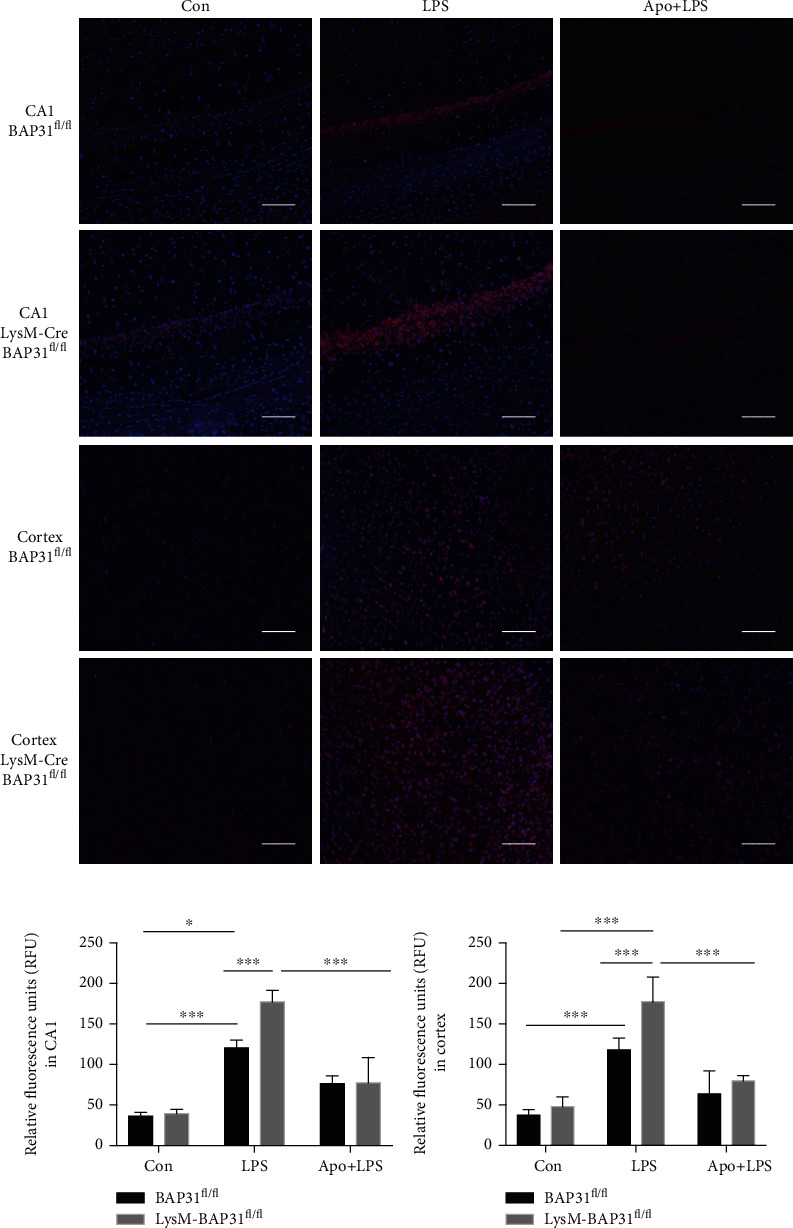
Apocynin prevents BAP31-deficiency-induced superoxide anion production *in vivo*. (a) Representative images of DHE staining from the hippocampus and cortex after LPS administration. Quantification of DHE staining of the hippocampus (b) and cortex (c) in BAP31^fl/fl^ and LysM-Cre-BAP31^fl/fl^ mice. Scale bars = 200 *μ*m. *n* = 8 per group for each experiment. Data are expressed as mean ± SEM. ^∗^*p* < 0.05, ^∗∗^*p* < 0.01, and ^∗∗∗^*p* < 0.001 versus the control group.

Recent studies have reported that intraperitoneal administration of LPS mediates superoxide anion accumulation [23]. BAP31^fl/fl^ and LysM-Cre-BAP31^fl/fl^ mice were intraperitoneally injected with LPS to assess the superoxide anion production in the brain. Consistently, we observed that systemic LPS administration enhanced the production of superoxide anion in both the BAP31^fl/fl^ and LysM-Cre-BAP31^fl/fl^ groups, but BAP31 deficiency significantly increased the accumulation of superoxide anion. As shown in Figure 8, BAP31 deficiency resulted in excessive superoxide production in the hippocampus (from 120.5 ± 3.39 to 177.1 ± 5.01, *F*_(2, 42)_ = 216.8, *p* < 0.001) and cortex (from 117.9 ± 5.121 to 177.4 ± 10.7, *F*_(2, 42)_ = 130.5, *p* < 0.001) after LPS administration compared with BAP31^fl/fl^ mice. Apocynin treatment significantly decreased superoxide production in the hippocampus (from 177.1 ± 5.013 to 77.47 ± 10.98, *F*_(2, 42)_ = 216.8, *p* < 0.001) and cortex (from 177.4 ± 10.7 to 79.91 ± 2.23, *F*_(2, 42)_ = 130.5, *p* < 0.001) in LysM-Cre-BAP31^fl/fl^ mice, consistent with the results using the BV2 cell line.

**Figure 9:**
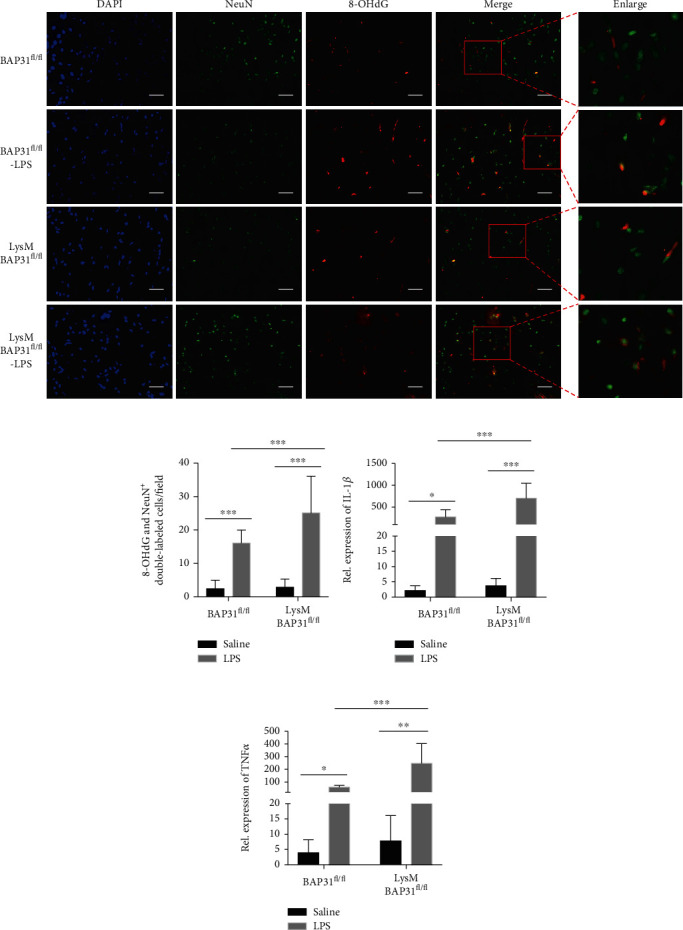
Conditional microglial BAP31 knockout mice exhibit more inflammation and oxidative damage when administered LPS. (a) A representative schematic diagram and microscopy images of 8-OHdG (green), NeuN (red), and DAPI (blue) triple immunofluorescent staining of the brain. Scale bars = 200 *μ*m. (b) Quantification of 8-OHdG (green) and NeuN (red) double positive cells. (c, d) Levels of IL-1*β* and TNF*α* mRNA in samples of the hippocampus were analyzed by RT-PCR. All the data are indicated as mean ± SEM of three independent experiments. ^∗^*p* < 0.05, ^∗∗^*p* < 0.01, and ^∗∗∗^*p* < 0.001 versus the control group.

To identify the location of oxidative damage in neurons, a triple immunofluorescent method with 8-OHdG (red fluorescence, DNA/RNA damage marker), NeuN (green fluorescence, a neuron cell marker), and DAPI (blue fluorescence, a nuclear dye) was employed. As depicted in the schematic (Figures 9(a) and 9(b)), in the LPS groups, 8-OHdG largely accumulated in the cytosol in NeuN-positive neurons. BAP31 deficiency increased 8-OHdG cytosolic accumulation.

To verify the function of BAP31 on inflammatory cytokines *in vivo*, we examined the production of the proinflammatory cytokines IL-1*β* and TNF*α* in the hippocampus of the mice described above by RT-PCR. LysM-Cre-BAP31^fl/fl^ mice administered LPS showed higher expression (Figures 9(c) and 9(d)) of the two cytokines compared with the BAP31^fl/fl^ mice (IL-1*β*: 275 ± 54.07-fold versus 700.8 ± 114.8-fold, *F*_(1, 32)_ = 58.39, *p* < 0.001; TNF*α*: 59.2 ± 4.81-fold versus 247.3 ± 51.8-fold). Consistent with the *in vitro* results, BAP31 deficiency significantly exacerbated cytokine production.

**Figure 10:**
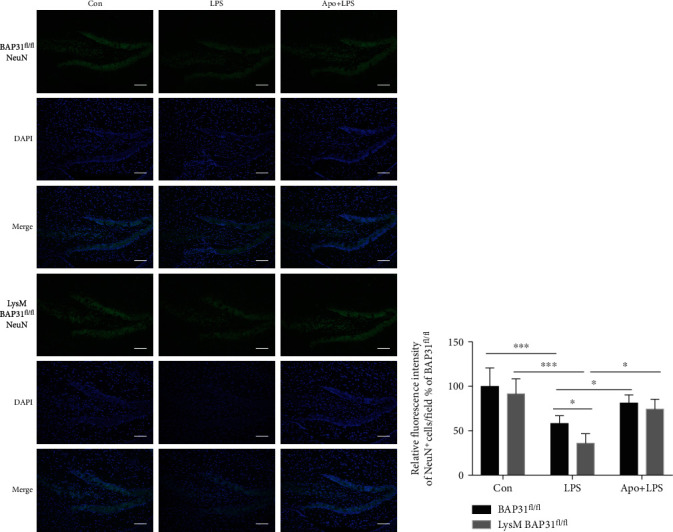
Apocynin protects hippocampal neurons from BAP31-deficiency-induced superoxide anion and inflammation *in vivo*. (a) Representative images of NeuN-labeled intact neurons in the hippocampal DG areas. The intact neuron is shown in green. Scale bars = 200 *μ*m. (b) Relative fluorescence intensity of NeuN+ cells in the DG. *n* = 8 per group in each experiment. Data are expressed as mean ± SEM. ^∗^*p* < 0.05, ^∗∗^*p* < 0.01, and ^∗∗∗^*p* < 0.001 versus the control group.

As BAP31 regulated superoxide anion and neuroinflammation, which are harmful to neurons, we examined the neuronal integrity using a NeuN antibody that detects intact neurons. Similar sections of the hippocampus were compared between the six groups of mice. As shown in Figure 10(a), NeuN-positive cells in the BAP31^fl/fl^ groups were densely packed in the DG region of the hippocampus, after LPS challenge, the total fluorescence intensity of NeuN-positive cells per field was significantly decreased in the DG regions, as indicated in Figure 10(b). BAP31 deficiency significantly decreased the fluorescence intensity of NeuN-positive cells compared with the BAP31^fl/fl^ mice from 67.24 ± 1.07% to 40.31 ± 1.10%, *F*_(2, 42)_ = 52.64, *p* < 0.001. Apocynin treatment significantly increased the fluorescence intensity of NeuN-positive cells in LysM-Cre-BAP31^fl/fl^ mice from 40.31 ± 1.10% to 93.19 ± 4.96%, *F*_(2, 42)_ = 52.64, *p* < 0.05.

## 4. Discussion

**Figure 11:**
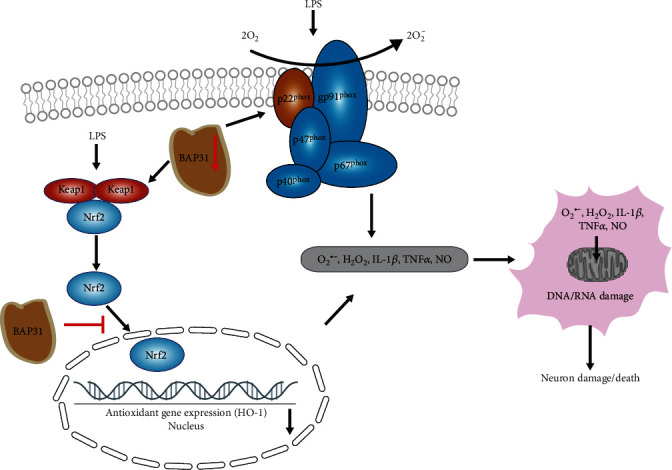
A schematic illustration of BAP31 regulating superoxide anion production and neuroinflammation in microglia. BAP31 deficiency upregulates LPS-induced superoxide anion and hydrogen peroxide production through p22^phox^ and the keap1/Nrf2/HO-1 signaling pathway, excess superoxide anion, and hydrogen peroxide cooperate with inflammatory cytokine to induce the damage and death of neurons.

Neurodegenerative diseases are characterized by chronic microglial overactivation and oxidative damage, wherein excessive levels of free radicals can overwhelm antioxidant response systems and lead to oxidative damage in the brain. Evidence indicates that superoxide anion and hydrogen peroxide are a major factor contributing to the initiation and/or progression of various neurodegenerative diseases [32]. As depicted in Figure 11, we found that BAP31 regulated LPS-induced superoxide anion and hydrogen peroxide production, and p22^phox^/keap1 knockdown or apocynin treatment inhibited superoxide anion production induced by BAP31 deficiency. Additionally, BAP31 regulated microglial superoxide anion and hydrogen peroxide production accompanied by alteration of inflammatory cytokine expression, which eventually led to oxidative damage and neuronal cell death.

The superoxide anion, an important free radical, is the source of other ROS. Hydrogen peroxide, a key reactive oxygen species, is produced at low levels during normal cellular metabolism and at higher concentrations under pathological conditions. An increased production of superoxide and hydrogen peroxide has been shown to mediate neuron death in neurodegenerative disease [33–35]. Therefore, our study examines the effects of superoxide anion and hydrogen peroxide in microglial cells. Under physiological circumstances, NOX likely generates only low levels of superoxide anion in the CNS [36, 37], and BAP31 deficiency had a weak effect on superoxide production. Under pathophysiological conditions, superoxide anion reacts irreversibly with several cellular constituents including protein phospholipids and nuclear DNA, causing lipid peroxidation [38, 39], membrane damage, dysregulation of cellular processes, and genome mutations [40–42]. We found that BAP31 deficiency upregulated LPS-induced superoxide and hydrogen peroxide production. Superoxide may be protonated to form hydroperoxyl radical or dismutated to form hydrogen peroxide, both of which may contribute to the initiation of lipid peroxidation [43–45]. Consistent with previous results, BAP31 deficiency exacerbated superoxide anion production following LPS treatment, resulting in lipid damage in microglia. Previous studies have shown that LPS leads to superoxide anion production by upregulating the expression of NOX in microglial cells [46, 47]. We also found that BAP31 deficiency upregulated LPS-induced superoxide anion production through the expression of NOX subunits, including p22^phox^, p40^phox^, p67^phox^, and gp91^phox^, indicating that BAP31 might regulate superoxide anion production via NOX. An increasing body of research suggests that gp91^phox^ forms a heterodimer with the smaller membrane-associated p22^phox^ protein, and together they make up the central component of NADPH oxidase, wherein p22^phox^ contributes to the maturation and stabilization of the heterodimer that it forms with gp91^phox^ [48]. As observed for Nox2, the Nox1, Nox3, and Nox4 proteins also form heterodimers with p22^phox^, which is essential for their activity, and p22^phox^ and gp91^phox^ immunoreactivity is observed almost exclusively on microglia in the CNS [49]. Our study showed that BAP31 upregulated the protein level of p22^phox^ and that knockdown of p22^phox^ reversed superoxide anion production and inflammatory cytokine release. Consistent with our findings, p22^phox^ mutation inhibits inflammatory oxidative damage in endothelial cells and vessels [50]. CRISPR/Cas9-mediated knockout of p22^phox^ leads to a loss of Nox1 and Nox4 activity. Downregulation of p22^phox^ ameliorates the inflammatory response during angiotensin II-induced oxidative stress by regulating MAPK and NF-*κ*B pathways in ARPE-19 cells [51], indicating that BAP31 regulates superoxide anion production by p22^phox^ and BAP31 has protective effects on the homeostasis in the CNS. NOX enzyme cytosolic subunits are typically in the cytoplasm in resting cells, but most NADPH oxidases are activity-dependent enzyme complexes and their activation usually requires the translocation of cytosolic subunits to the membrane-bound subunits p22^phox^ and NOX isoforms. Under pathophysiological conditions, NOX enzyme cytosolic subunits translocate to membranes in response to cellular activation. Upregulated p22^phox^ promotes the further activation of NOX and results in a massive increase in superoxide anion. In this study, we discovered that lack of BAP31 increases the expression level of p22^phox^. However, the relative changes between BAP31 deficiency and scramble cells showed no significant differences after LPS treatment, indicating that the sensitivity of p22^phox^ upregulation to LPS could be reduced as a result of BAP31 deficiency. Alternatively, the relative expression levels of those indicators that were not affected by BAP31 deficiency increased after the LPS treatment.

Keap1 is a cysteine-based mammalian intracellular sensor for electrophiles and oxidants [52]. The keap1-Nrf2 system plays important roles in the antioxidant response and contributes to cytoprotection from various redox disturbances [53]. Our study found that BAP31 deficiency upregulated the expression of keap1 and inhibited the translocation of Nrf2; knockdown of keap1 reversed superoxide anion production and inflammatory cytokine release caused by BAP31 deficiency by reversing the translocation of Nrf2. Similar to our results, repression of keap1 expression increases Nrf2 activity [54], and Nrf2 activation ameliorates inflammation and tissue damage in a sickle cell model [55], indicating that BAP31 may also regulate superoxide anion production via the keap1/Nrf2/HO-1 signaling pathway and that the reversal of keap1/Nrf2/HO-1 signaling might alleviate the superoxide anion production induced by BAP31 deficiency.

It is beginning to be recognized that superoxide anion polarizes microglia toward a classical proinflammatory phenotype, and excess superoxide anion accelerates the inflammatory response [56]. Here, we found that BAP31 deficiency exacerbated superoxide anion and proinflammatory cytokine production and decreased superoxide anion production by p22^phox^/keap1 silencing in BAP31-deficient cells alleviated inflammatory cytokine production, indicating that BAP31 influenced inflammatory cytokine production through superoxide anion production.

A previous study has reported that microglial activation leads to noxious effects on neurons and participates in the pathophysiology of neurodegenerative diseases [57]. A key determinant of microglial neurotoxicity is the release of excitotoxins, including superoxide anion and inflammatory cytokines [58]. Consistent with the results of previous studies, our conditioned coculture *in vitro* studies indicated that superoxide anion and the inflammatory cytokines under conditions of BAP31 deficiency exacerbated the damage and death in neurons. *In vivo*, we applied the Cre-LoxP system to produce conditional BAP31 knockdown mice, and our results showed that BAP31 deficiency exacerbated neuronal oxidative damage and death by exacerbating superoxide anion and inflammatory cytokine production *in vivo*. Upon treatment with apocynin, the survival rate of neurons was elevated *in vitro* and *in vivo*, which is consistent with a report showing that apocynin prevents learning and memory deficits by protecting the neurons from superoxide and inflammatory cytokines [25, 43, 59, 60]. These results indicate that apocynin has a therapeutic effect on neuroinflammation caused by BAP31 deficiency, and BAP31 might be an important regulator of superoxide anion production and neuroinflammation.

In summary, we illustrated that BAP31 deficiency may contribute to chronic unremitting inflammation in neurodegenerative diseases by promoting superoxide anion and inflammatory cytokine production, indicating that BAP31 might be a key regulator of microglial-related inflammation and neurotoxicity.

## 5. Conclusions

The present results suggest that BAP31 deficiency contributes to microglia-related superoxide anion production through p22^phox^ and keap1 pathways. The excess superoxide anion and inflammatory cytokines work together to induce damage and death in neurons. These studies highlight that BAP31 is an important regulator of superoxide anion production and neuroinflammation, and as such, the downstream regulators or agonists of BAP31 could be considered as a potential therapeutic targets in microglia-related superoxide anion production and neuroinflammation.

## Data Availability

The data used to support the findings of this study are available from the corresponding authors upon request.

## Abbreviation

AD: Alzheimer's disease
BAP31: B cell receptor-associated protein 31
CNS: Central nervous system
MCM: Microglia conditional medium
DHE: Dihydroethidium
H₂DCFDA: 2′,7′-Dichloride hydro-fluorescein diacetate
ER: Endoplasmic reticulum
ELISA: Enzyme-linked immune sorbent assay
HO-1: Heme oxygenase-1
keap1: Kelch-like ECH-associated protein 1
LPS: Lipopolysaccharide
MS: Multiple sclerosis
NOX: NADPH oxidase
Nrf2: Nuclear factor erythroid 2-related factor 2
NBT: 4-Nitro blue tetrazolium chloride
PD: Parkinson's disease
ROS: Reactive oxygen species
RT-PCR: Reverse transcription quantitative real-time polymerase chain reaction.

## Supplementary Materials

Supplementary Materials Supplementary Figure 1: BAP31 deficiency exacerbates LPS-induced superoxide anion generation in microglia. (a, b) Scramble and shBAP31 cells were stimulated with LPS (100 ng/ml) for 12 h. Superoxide anion was measured by staining with DHE and observed by confocal microscopy. The relative fluorescence intensity detected by DHE staining in (a) was quantified by ImageJ. Scale bars = 100 *μ*m. All the data are indicated as mean ± SEM of three independent experiments. ^∗^*p* < 0.05, ^∗∗^*p* < 0.01, and ^∗∗∗^*p* < 0.001 versus the control group. Supplementary Figure 2: p47^phox^ was not influenced by BAP31. Scramble and shBAP31 BV2 cells were treated with LPS (100 ng/ml) for 24 h. The mRNA levels of p47^phox^ were analyzed with RT-PCR. All the data are indicated as mean ± SEM of three independent experiments. ^∗^*p* < 0.05, ^∗∗^*p* < 0.01, and ^∗∗∗^*p* < 0.001 versus the control group. Supplementary Figure 3: apocynin prevents NADPH oxidase production in shBAP31 microglial cells. Scramble and shBAP31 BV2 cells were treated with apocynin for 1 h and then stimulated with LPS (100 ng/ml) for 24 h. The mRNA levels of the p22^phox^ (a), p40^phox^ (b), p67^phox^ (c), and gp91^phox^ (d) were analyzed with RT-PCR. All the data are indicated as mean ± SEM of three independent experiments. ^∗^*p* < 0.05, ^∗∗^*p* < 0.01, and ^∗∗∗^*p* < 0.001 versus the control group. Supplementary Figure 4: apocynin alleviates superoxide anion production caused by the deficiency of BAP31. (a, b) Primary microglial cells from BAP31^fl/fl^ and LysM-Cre-BAP31^fl/fl^ mice were pretreated with apocynin for 1 h and then stimulated with LPS (100 ng/ml) for 12 h. Superoxide anion was measured by staining with DHE and observed by confocal microscopy. The relative fluorescence intensity was quantified by ImageJ. Scale bars = 100 *μ*m. All the data are indicated as mean ± SEM of three independent experiments. ^∗^*p* < 0.05, ^∗∗^*p* < 0.01, and ^∗∗∗^*p* < 0.001 versus the control group. Supplementary Figure 5: apocynin prevents proinflammatory cytokine production in BV2 microglial cells. Scramble and shBAP31 BV2 cells were pretreated with apocynin for 1 h and then stimulated with LPS (100 ng/ml) for 24 h. The mRNA levels of BAP31 (a), IL-1*β* (b), TNF*α* (c), CD80 (d), CD86 (e), Arg1 (f), CD206 (g), Fizz1 (h), and Ym1 (i) were analyzed with RT-PCR. All the data are indicated as mean ± SEM of three independent experiments. ^∗^*p* < 0.05, ^∗∗^*p* < 0.01, and ^∗∗∗^*p* < 0.001 versus the control group.
