# Supplementary material for: Regulation of Superoxide by BAP31 through Its Effect on p22phox and Keap1/Nrf2/HO-1 Signaling Pathway in Microglia
Source: Oxid Med Cell Longev. 2021 Mar 9;2021:1457089. doi: 10.1155/2021/1457089 (PMC7969104; doi:10.1155/2021/1457089)
Supplement: Supplementary Materials — Supplementary Figure 1: BAP31 deficiency exacerbates LPS-induced superoxide anion generation in microglia. (a, b) Scramble and shBAP31 cells were stimulated with LPS (100 ng/ml) for 12 h. Superoxide anion was measured by staining with DHE and observed by confocal microscopy. The relative fluorescence intensity detected by DHE staining in (a) was quantified by ImageJ. Scale bars = 100 μm. All the data are indicated as mean ± SEM of three independent experiments. ∗p < 0.05, ∗∗p < 0.01, and ∗∗∗p < 0.001 versus the control group. Supplementary Figure 2: p47phox was not influenced by BAP31. Scramble and shBAP31 BV2 cells were treated with LPS (100 ng/ml) for 24 h. The mRNA levels of p47phox were analyzed with RT-PCR. All the data are indicated as mean ± SEM of three independent experiments. ∗p < 0.05, ∗∗p < 0.01, and ∗∗∗p < 0.001 versus the control group. Supplementary Figure 3: apocynin prevents NADPH oxidase production in shBAP31 microglial cells. Scramble and shBAP31 BV2 cells were treated with apocynin for 1 h and then stimulated with LPS (100 ng/ml) for 24 h. The mRNA levels of the p22phox (a), p40phox (b), p67phox (c), and gp91phox (d) were analyzed with RT-PCR. All the data are indicated as mean ± SEM of three independent experiments. ∗p < 0.05, ∗∗p < 0.01, and ∗∗∗p < 0.001 versus the control group. Supplementary Figure 4: apocynin alleviates superoxide anion production caused by the deficiency of BAP31. (a, b) Primary microglial cells from BAP31fl/fl and LysM-Cre-BAP31fl/fl mice were pretreated with apocynin for 1 h and then stimulated with LPS (100 ng/ml) for 12 h. Superoxide anion was measured by staining with DHE and observed by confocal microscopy. The relative fluorescence intensity was quantified by ImageJ. Scale bars = 100 μm. All the data are indicated as mean ± SEM of three independent experiments. ∗p < 0.05, ∗∗p < 0.01, and ∗∗∗p < 0.001 versus the control group. Supplementary Figure 5: apocynin prevents proinflammatory cytokine production in BV2 microgl [file 1457089.f1.docx]

Supplementary Materials


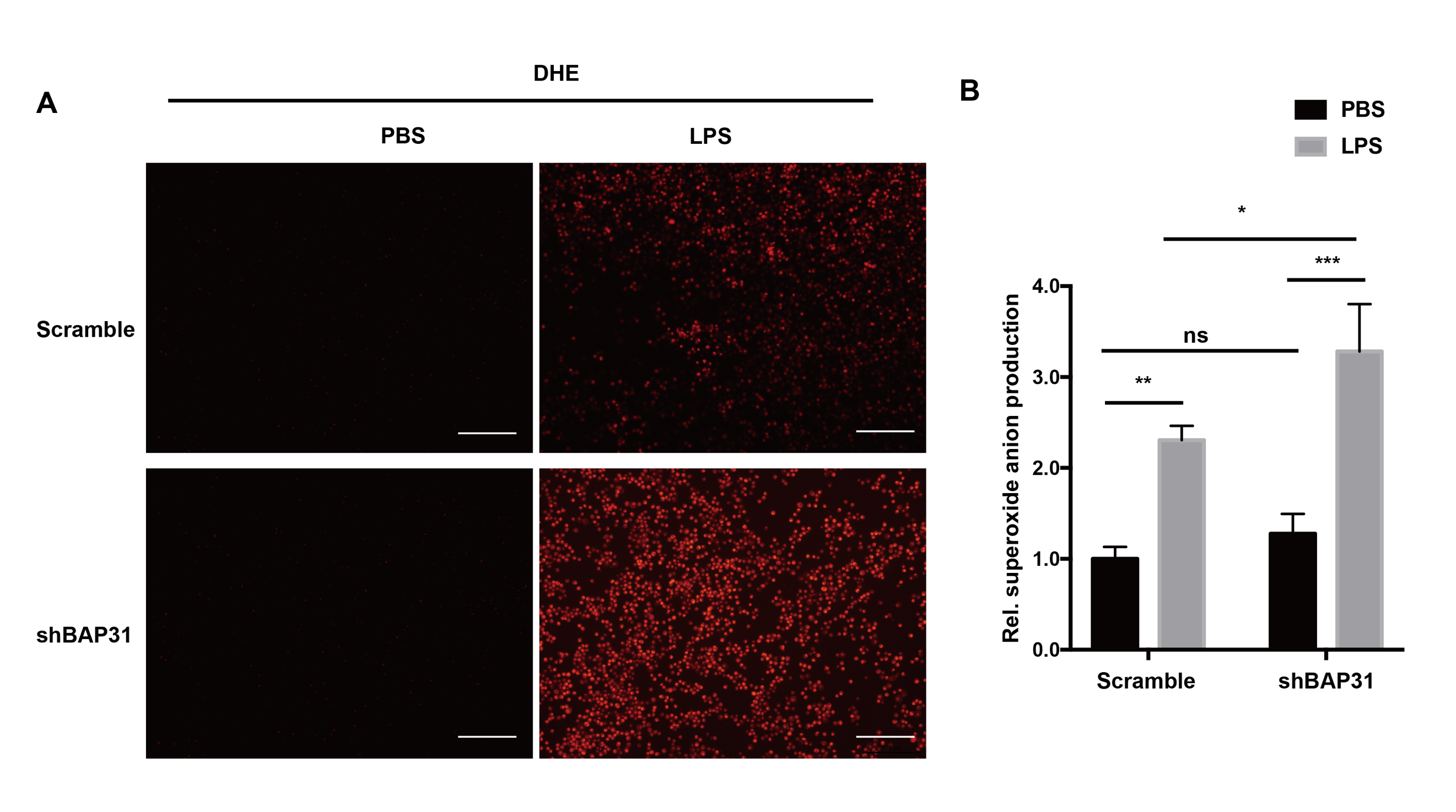


Supplementary Fig. 1 BAP31 deficiency exacerbates LPS-induced superoxide anion generation in microglia. (a, b) Scramble and shBAP31 cells were stimulated with LPS (100 ng/ml) for 12 h. superoxide anion were measured by staining with DHE and observed by confocal microscopy. The relative fluorescence intensity detected by DHE staining in (a) was quantified by ImageJ. Scale bars = 100 μm. All the data are indicated as mean ± SEM of three independent experiments. *P<0.05, **P<0.01, ***P<0.001 versus control group.


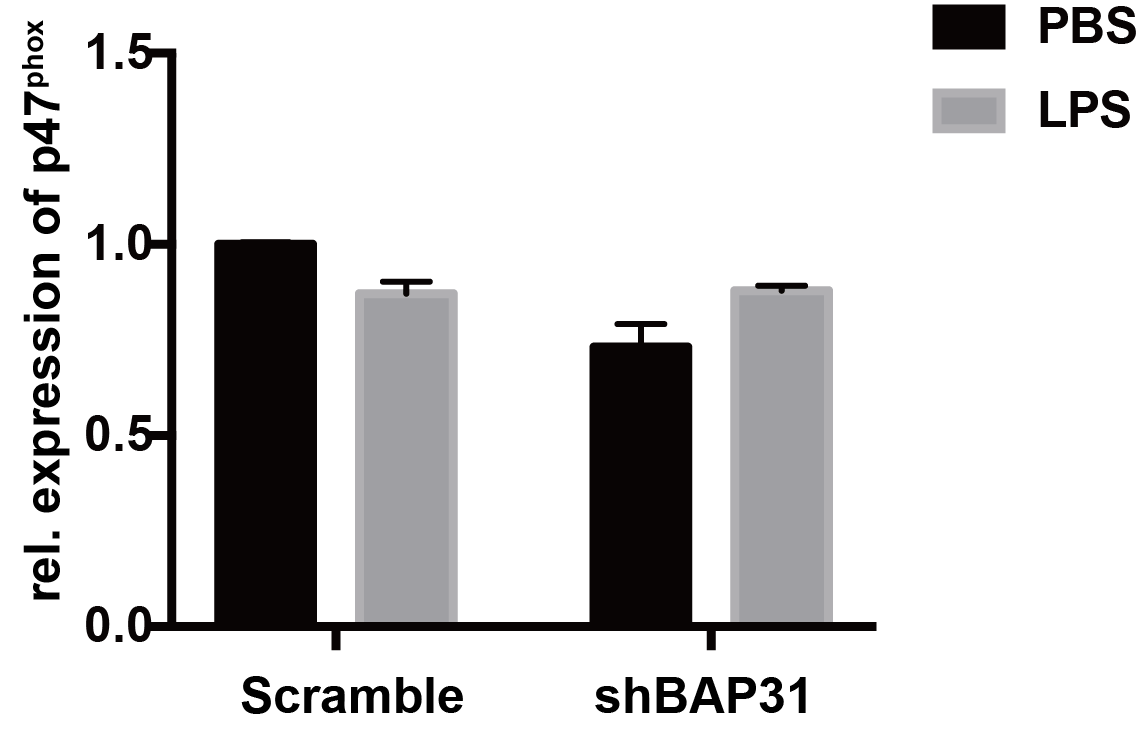


Supplementary Fig. 2 p47^phox^ was not influenced by BAP31. Scramble and shBAP31 BV2 cells were treated with LPS (100 ng/ml) for 24 h. The mRNA levels of the p47^phox^ was analyzed with RT-PCR. All the data are indicated as mean ± SEM of three independent experiments. *P<0.05, **P<0.01, ***P<0.001 versus control group.


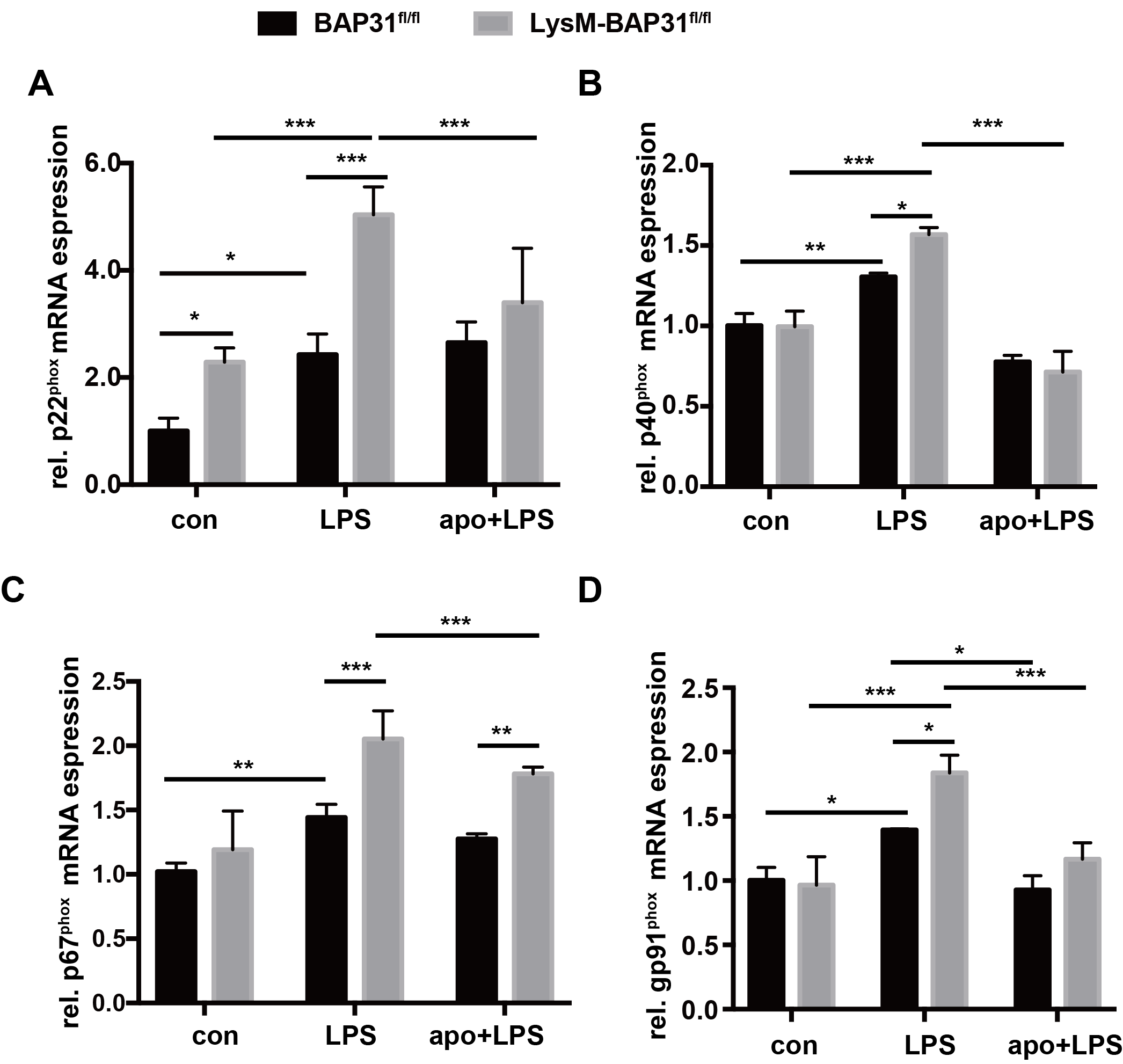


Supplementary Fig. 3 Apocynin prevents NADPH oxidase production in shBAP31 microglial cells. Scramble and shBAP31 BV2 cells were treated with apocynin for 1 h and then stimulated with LPS (100 ng/ml) for 24 h. The mRNA levels of the p22^phox^ (a), p40^phox^ (b), p67^phox^ (c) and gp91^phox^ (d) were analyzed with RT-PCR. All the data are indicated as mean ± SEM of three independent experiments. *P<0.05, **P<0.01, ***P<0.001 versus control group.


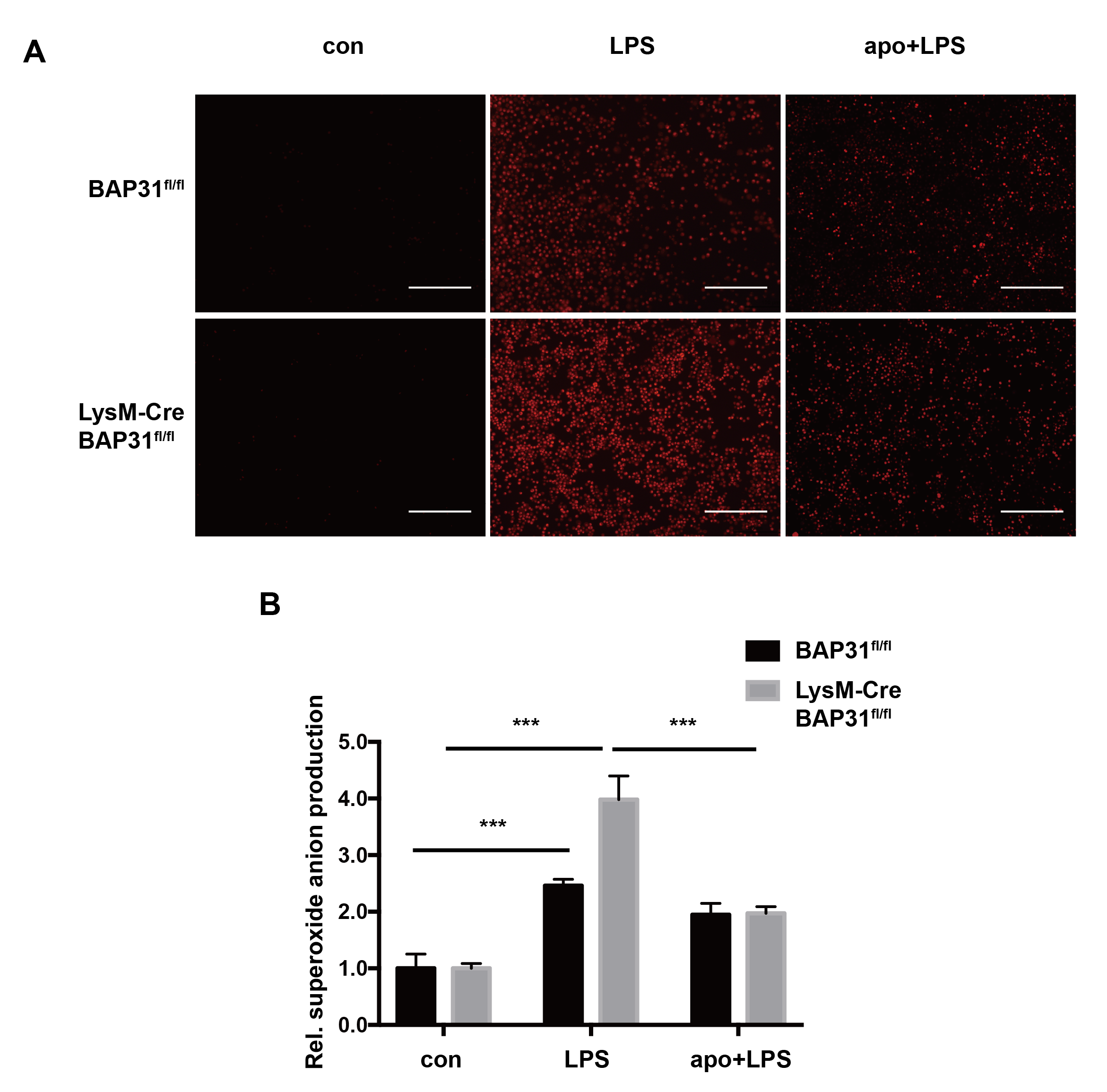


Supplementary Fig. 4 Apocynin alleviates superoxide anion production caused by the deficiency of BAP31. (a, b) Primary microglial cells from BAP31^fl/fl^ and LysM-Cre-BAP31^fl/fl^ mice were pretreated with apocynin for 1 h and then stimulated with LPS (100ng/ml) for 12 h. superoxide anion were measured by staining with DHE and observed by confocal microscopy. The relative fluorescence intensity was quantified by ImageJ. Scale bars = 100 μm. All the data are indicated as mean ± SEM of three independent experiments. *P<0.05, **P<0.01, ***P<0.001 versus control group.


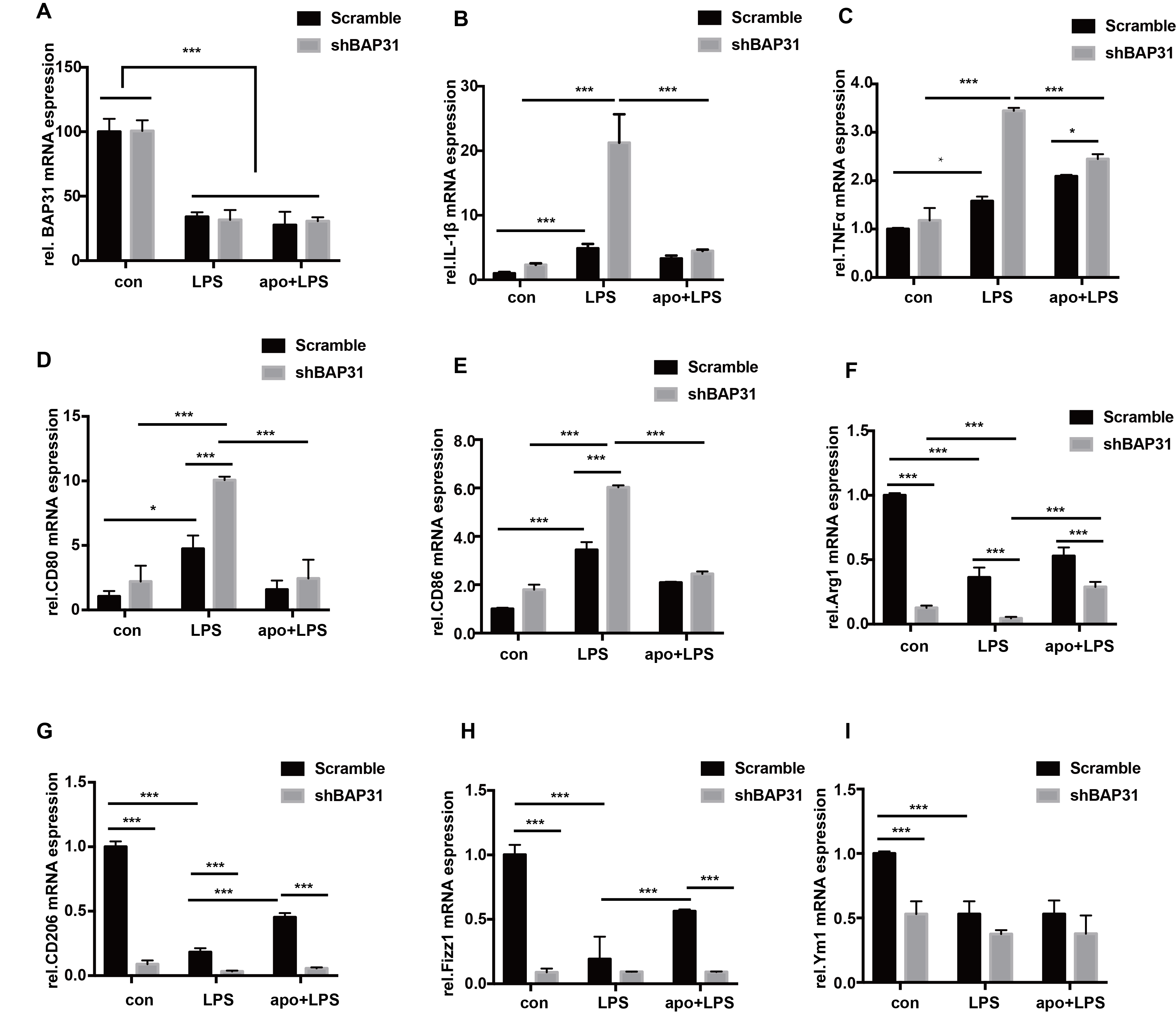


Supplementary Fig. 5 Apocynin prevents proinflammatory cytokines production in BV2 microglial cells. Scramble and shBAP31 BV2 cells were pretreated with apocynin for 1 h and then stimulated with LPS (100 ng/ml) for 24 h. The mRNA levels of the BAP31(a), IL-1β (b), TNFα (c), CD80 (d), CD86 (e), Arg1(f), CD206 (g), Fizz1(h) and Ym1(i) were analyzed with RT-PCR. All the data are indicated as mean ± SEM of three independent experiments. *P<0.05, **P<0.01, ***P<0.001 versus control group.
